# Supplementary material for: Harnessing insulin- and leptin-induced oxidation of PTP1B for therapeutic development
Source: Nat Commun. 2018 Jan 18;9:283. doi: 10.1038/s41467-017-02252-2 (PMC5773487; doi:10.1038/s41467-017-02252-2)
Supplement: Supplementary file 1 — Supplementary Information [file 41467_2017_2252_MOESM1_ESM.pdf]

Table 1 Data collection and refinement statistics

	scFv45
**Data collection**	
Space group	P 3_2 2 1
Cell dimensions	
a, b, c (Å)	97.23 97.23 61.26
α, β, γ (°)	90, 90, 120
Resolution (Å)	42.1–1.64 (1.69-1.64)
R_{merge}	0.049 (0.442)
R_{meas}	0.058 (0.555)
$CC_{1/2}$	0.998 (0.759)
CC^*	1 (0.929)
I/σ (I)	17.33 (2.56)
Completeness (%)	89.93 (83.52)
Redundancy	3.2 (2.6)
**Refinement**	
Resolution (Å)	1.64
No. reflections	37403
R_{work}/R_{free}	0.172/0.196
No. atoms	
Protein	1640
B factors (Å^2)	
Protein	12.75
Water	26.97
R.m.s deviations	
Bond lengths (Å)	0.006
Bond angles (°)	0.81

Structure was determined from one crystal. Values in parentheses are for the highest-resolution shell.

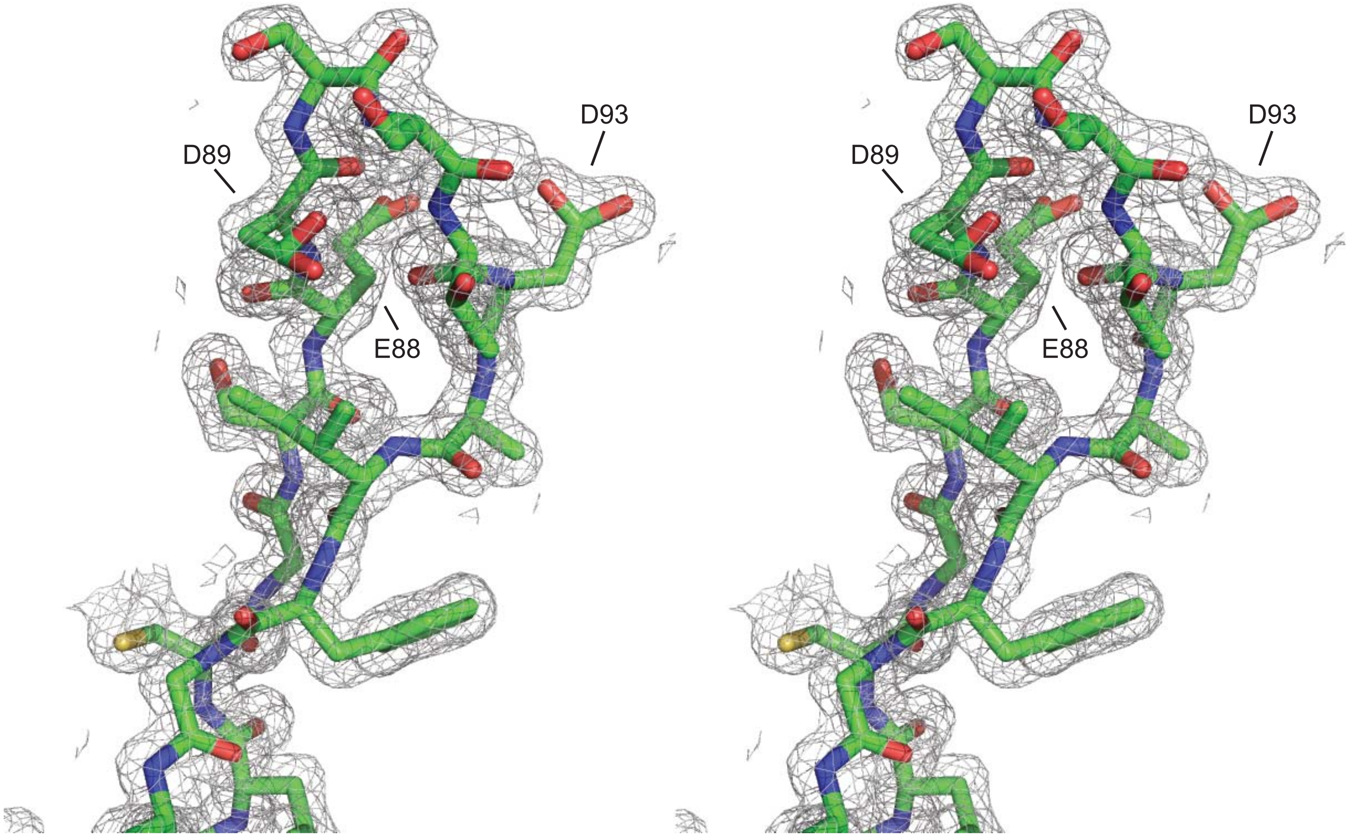

Representative stereo image of the electron density map of scFv45. The 2FO-FC electron density map of scFv45 calculated after the final refinement contoured at 1.0 σ. Residues from CDR region shown in stick representation. Figure generated by PyMol.

Supplementary Table 1

a

PTP1B-CASA - AspN Digestion

m/z (mi)	*m/z* (av)	Start	End	Sequence
827.4006	827.8772	22	28	(Q) DIRHEAS(D)
1171.538	1172.247	289	297	(Q) DQWKELSHE(D)
1262.660	1263.407	53	62	(F) DHSRIKLHQE(D)
2067.186	2068.488	300	317	(L) EPPPEHIPPPPRPPKRIL(E)
2295.297	2296.738	298	317	(E) DLEPPPEHIPPPPRPPKRIL(E)
2375.299	2376.812	29	47	(S) DFPCRVAKLPKNKNRNRYR(D)
2520.142	2521.845	1	21	(-) MEMEKEFEQIDKSGSWAAIYQ(D)

PTP1B-CASA-mut2 - AspN Digestion

1560.695	1561.632	318	329	(L) EPHNLEHHHHHH(-)

b

PTP1B-CASA - LysC Digestion

m/z (mi)	*m/z* (av)	Start	End	Sequence
878.4843	879.0125	315	321	(K) RILEPHN(-)
1518.824	1519.883	104	116	(K) SRGVVMLNRVMEK(G)
1556.697	1557.793	1	12	(-) MEMEKEFEQIDK(S)
1772.881	1773.952	59	73	(K) LHQEDNDYINASLIK(M)
2160.117	2161.406	42	58	(K) NRNRYRDVSPFDHSRIK(L)
2402.255	2403.685	40	58	(K) NKNRNRYRDVSPFDHSRIK(L)
2508.288	2509.842	293	314	(K) ELSHEDLEPPPEHIPPPPRPPK(R)
3045.536	3047.487	13	39	(K) SGSWAAIYQDIRHEASDFPCRVAKLPK(N)
3287.674	3289.767	13	41	(K) SGSWAAIYQDIRHEASDFPCRVAKLPKNK(N)
3367.755	3369.832	293	321	(K) ELSHEDLEPPPEHIPPPPRPPKRILEPHN(-)

Internal Peptide Standards

1296.685	1297.510	1	10	(-) DRVYIHPFHL(-)
2093.087	2094.460	1	17	(-) SYSMEHFRWGKPVGKKR(R)
2465.199	2466.720	18	39	(R) RPVKVYPNGAEDESAEAFPLEF(-)
3657.929	3660.190	7	38	(H) FRWGKPVGKKRRPVKVYPNGAEDESAEAFPLE(F)

In silico peptide digest masses for PTP1B-CASA : scFv45 protease protection assays. a) Monoisotopic (mi) and average (av) peptide mass-to-charge (m/z) ratio, peptide start and end residue numbers, and peptide amino acid sequence for PTP1B-CASA and PTP1B-CASA-mut2 in silico AspN digestion. b) Monoisotopic (mi) and average (av) peptide mass-to-charge (m/z) ratio, peptide start and end residue numbers, and peptide amino acid sequence for PTP1B-CASA in silico LysC digestion and internal peptide standards.

Supplementary Figure 1

a

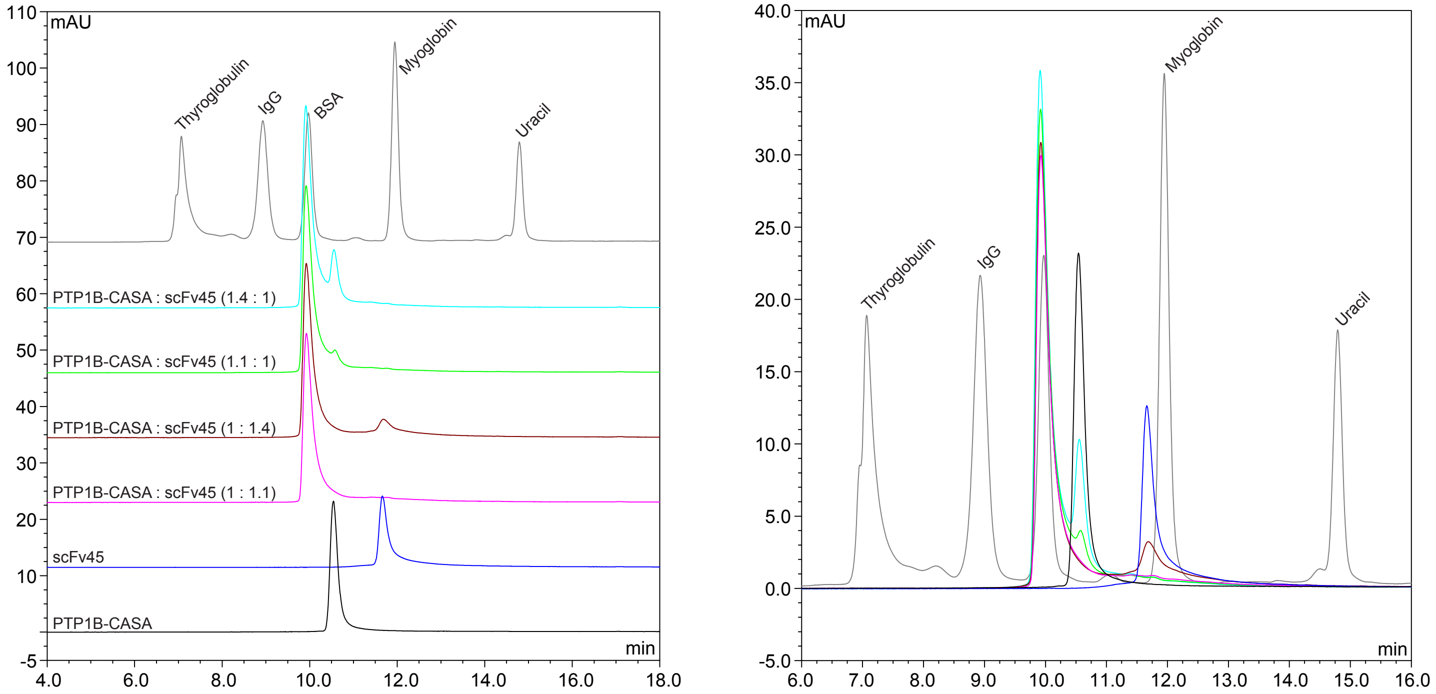

b

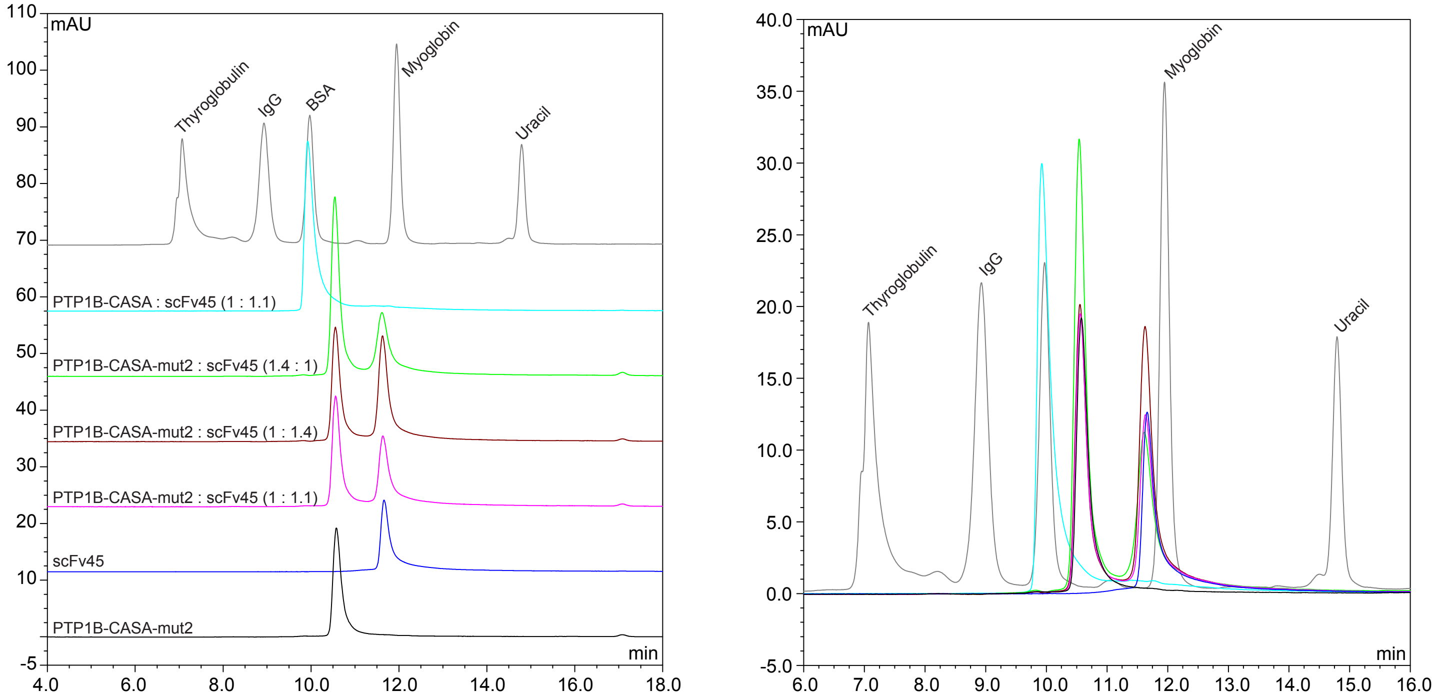

Oxidation-induced conformation of PTP1B requires the amino-terminal residues Leu37, Lys39, and Lys41 for scFv45 binding. a) Analytical size-exclusion chromatography of PTP1B-CASA in the absence or presence of scFv45 (at indicated molar ratios). b) Analytical size-exclusion chromatography of PTP1B-CASA-mut2 in the absence or presence of scFv45 (at indicated molar ratios). All data are representative of at least two independent experiments.

Supplementary Figure 2

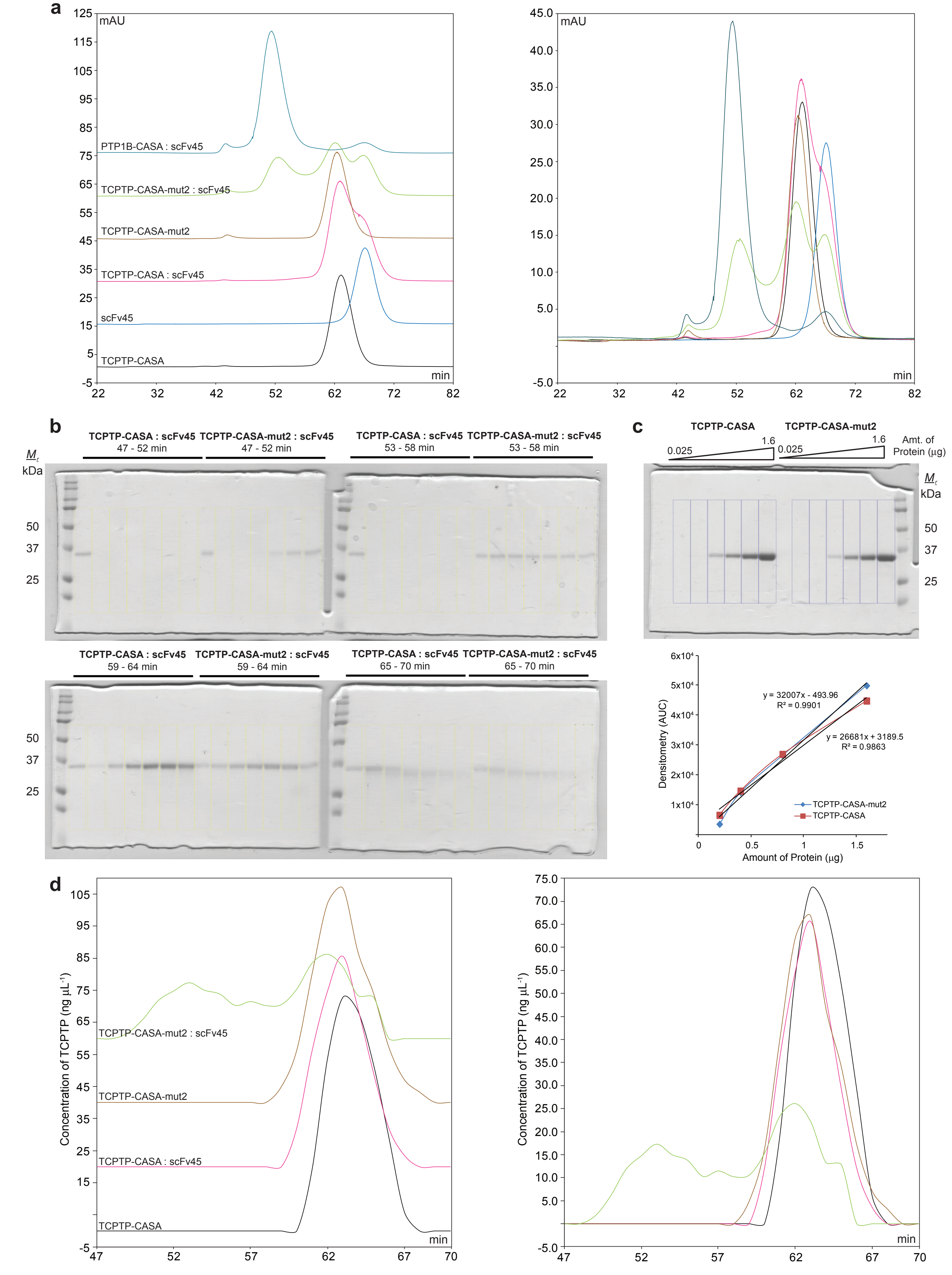

Adopting the amino-terminal residues Leu37, Lys39, and Lys41 of PTP1B enables gain-of-association between the oxidation-induced conformation of TCPTP and scFv45. a) Size-exclusion chromatography in the absence (TCPTP-CASA and TCPTP-CASA-mut2) or presence (TCPTP-CASA, TCPTP-CASA-mut2, and PTP1B-CASA) of scFv45 at 1.1-fold molar excess. b) SDS-PAGE and densitometry analysis of the indicated protein fractions from TCPTP-CASA : scFv45 and TCPTP-CASA-mut2 : scFv45 complexes separated by size-exclusion chromatography. Protein molecular weight standard (lane 1) and internal loading controls for TCPTP-CASA and TCPTP-CASA-mut2 (0.2 µg each; lanes 2 and 9 respectively) were included in all gels to normalize protein staining. c) SDS-PAGE, densitometry analysis, and standard curve generation from known amounts of TCPTP-CASA and TCPTP-CASA-mut2. d) Absolute concentration chromatogram of TCPTP-CASA or TCPTP-CASA-mut2 generated from converted densitometry measurements (b) using appropriate standard curve (c). All data are representative of at least two independent experiments.

Supplementary Figure 3

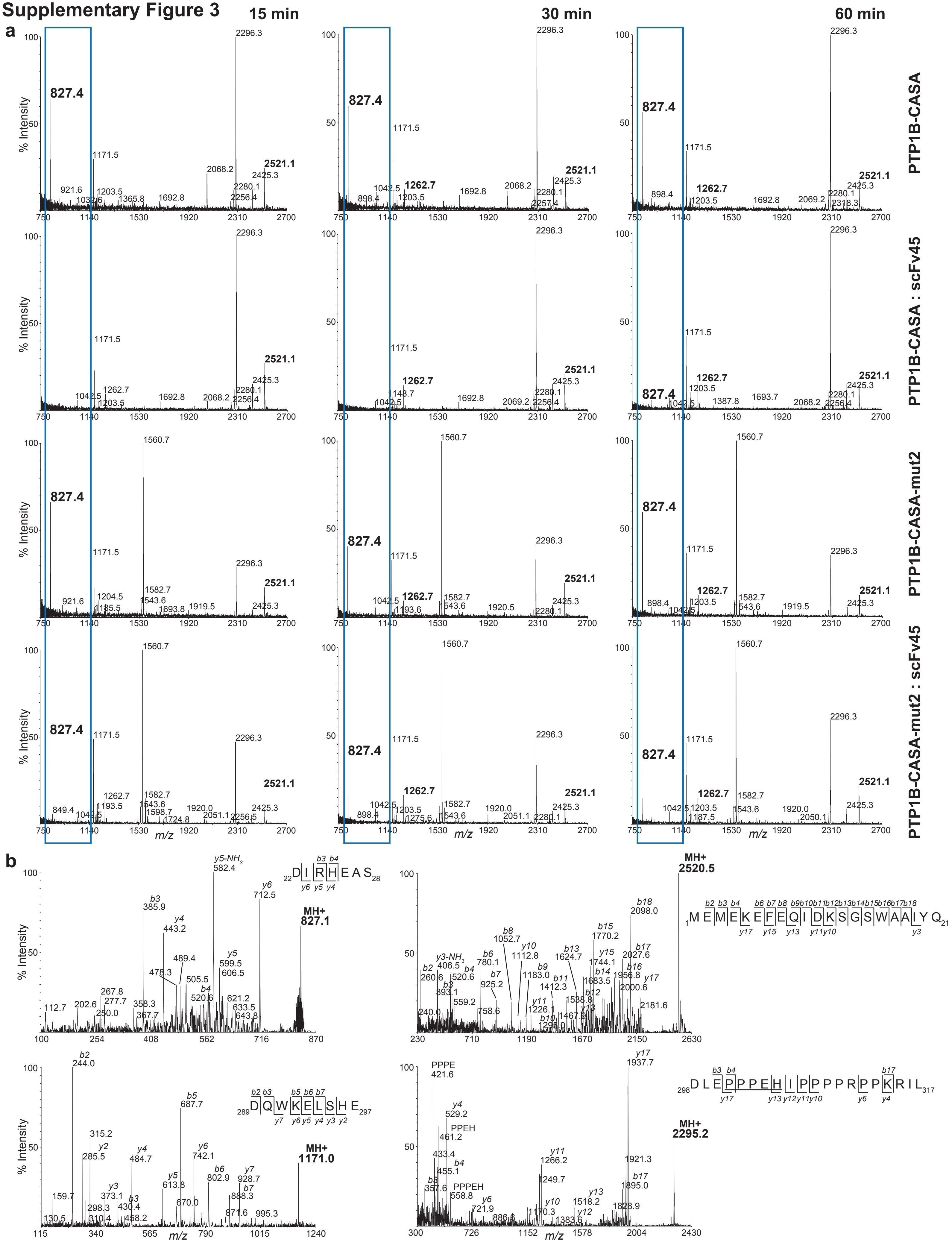

Oxidation-induced conformation of PTP1B requires the residues Leu37, Lys39, and Lys41 for proteolytic protection of the amino-terminal scFv45 binding interface. a) Mass spectra of PTP1B-CASA and PTP1B-CASA-mut2 in the absence or presence of 1.1-fold molar excess scFv45, digested with AspN endoprotease (1:500 w/w) for the indicated times. Peptide masses defining regional boundaries of PTP1B-CASA proteolytic protection are shown in **bold**, with those directly protected being highlighted (□). b) Tandem mass spectra (MS/MS) of target PTP1B-CASA and PTP1B-CASA-mut2 peptide ions from AspN protease protection assay. All data are representative of at least two independent experiments.

Supplementary Figure 4

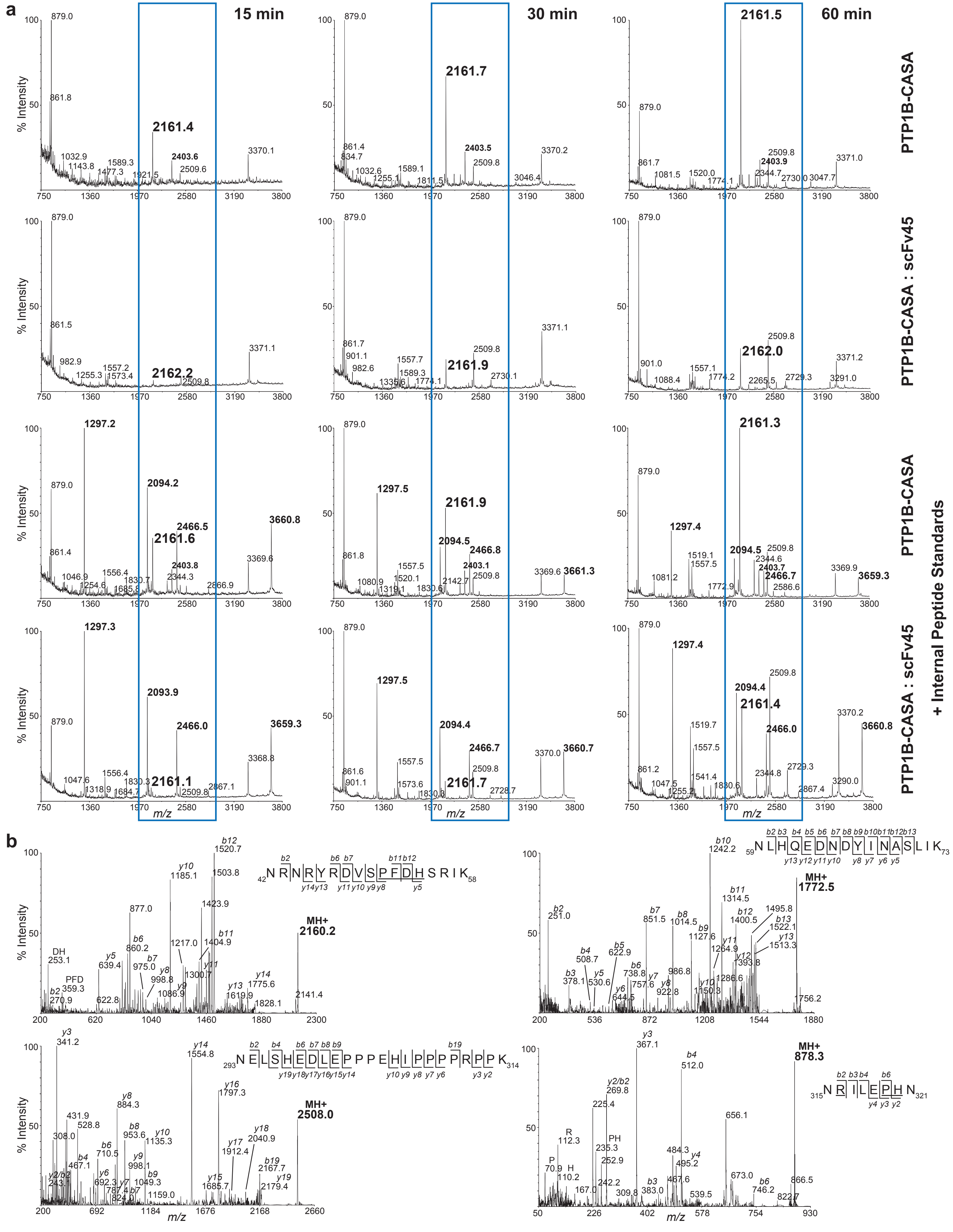

Oxidation-induced conformation of PTP1B confers proteolytic protection of the amino-terminal residues Lys39 and Lys41 upon scFv45 binding. a) Mass spectra of PTP1B-CASA in the absence or presence of 1.1-fold molar excess scFv45, digested with LysC endoprotease (1:750 w/w) for the indicated times, in the absence or presence of an approximate average of 42.5 fmol internal peptide standards as indicated. Masses of internal peptide standards are shown in **bold**, with those peptide masses of PTP1B-CASA directly protected from proteolysis being highlighted (□) in **bold**. b) Tandem mass spectra (MS/MS) of target PTP1B-CASA peptide ions from LysC protease protection assay. All data are representative of at least two independent experiments.

Supplementary Figure 5

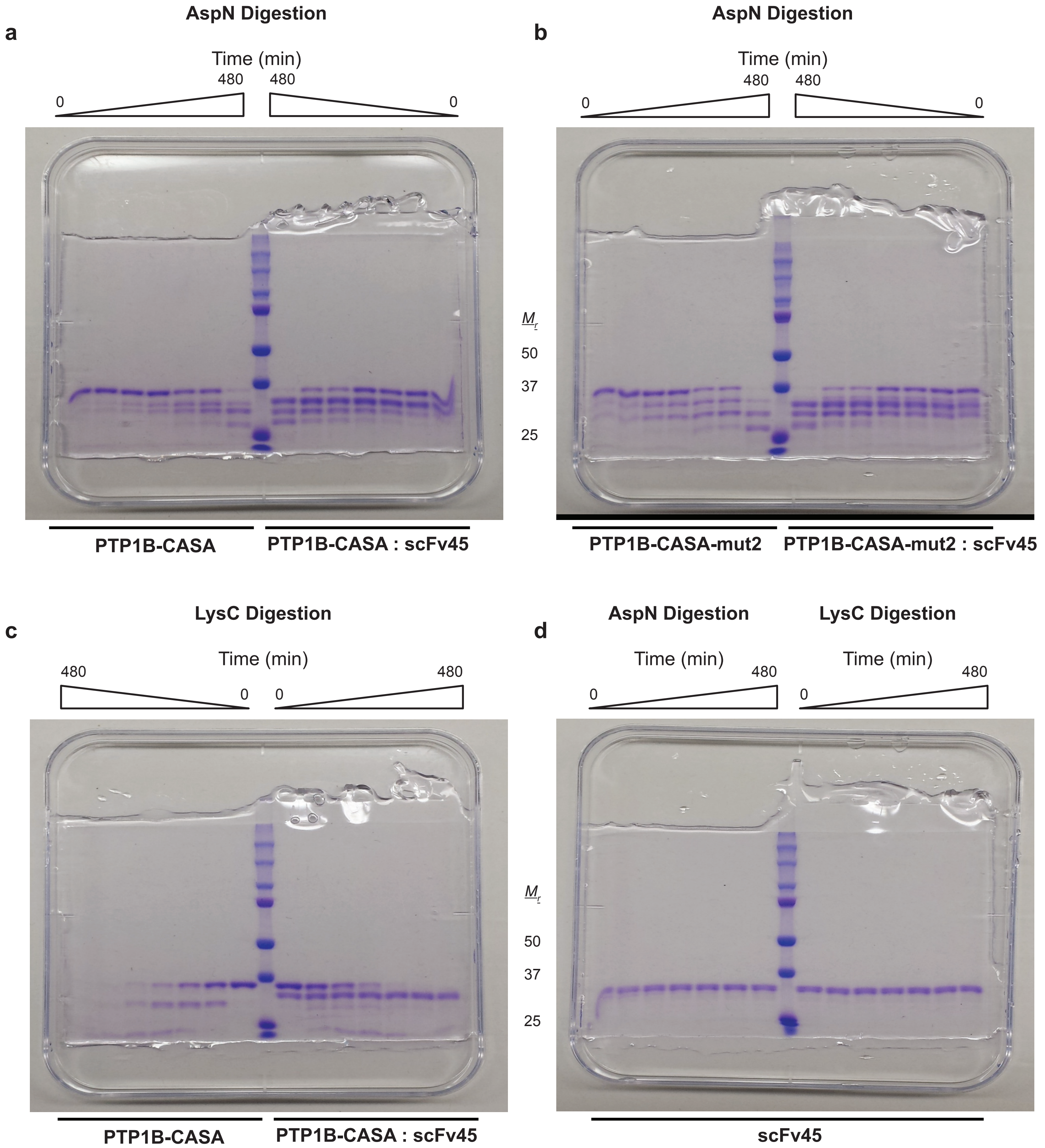

Oxidation-induced conformation of PTP1B requires the amino-terminal residues Leu37, Lys39, and Lys41 for scFv45 binding and proteolytic protection. a) SDS-PAGE of PTP1B-CASA in the absence or presence of 1.1-fold molar excess scFv45, digested with AspN endoprotease (1:500 w/w) for the indicated times. b) SDS-PAGE of PTP1B-CASA-mut2 in the absence or presence of 1.1-fold molar excess scFv45, digested with AspN endoprotease (1:500 w/w) for the indicated times. c) SDS-PAGE of PTP1B-CASA in the absence or presence of 1.1-fold molar excess scFv45, digested with LysC endoprotease (1:750 w/w) for the indicated times. d) SDS-PAGE of scFv45 digested with AspN (1:500 w/w) or LysC endoprotease (1:750 w/w) for the indicated times. All data are representative of at least two independent experiments.

Supplementary Figure 6

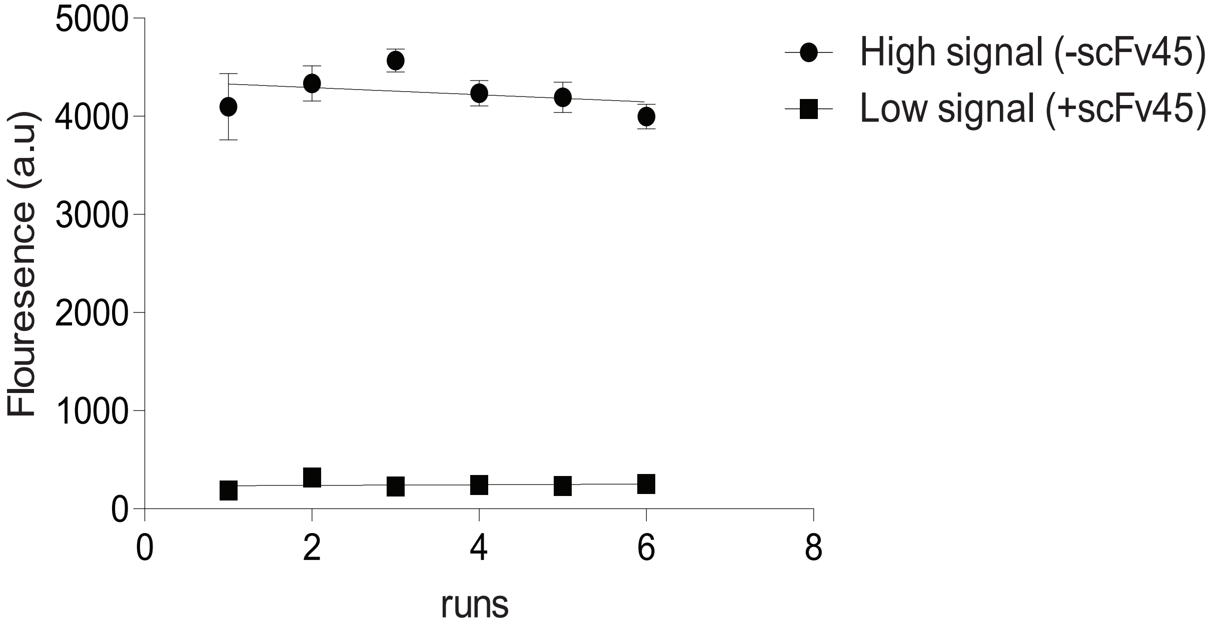

Validation of the screening assay to identify small molecule inhibitors that mimic the effects of the oxidation sensor scFvs on PTP1B. Reversibly oxidized PTP1B (PTP1B-OX) was generated by incubation with H_2O_2 in vitro, desalted, then assayed (20 nM) in the presence and absence of scFv45 (100 nM) using DiFMUP as substrate. The assay was performed multiple times to calculate the Z-prime value. Data points represent the mean ± s.e.m. from three individual replicates each arising from an independent experimental run (x-axis).

Supplementary Figure 7

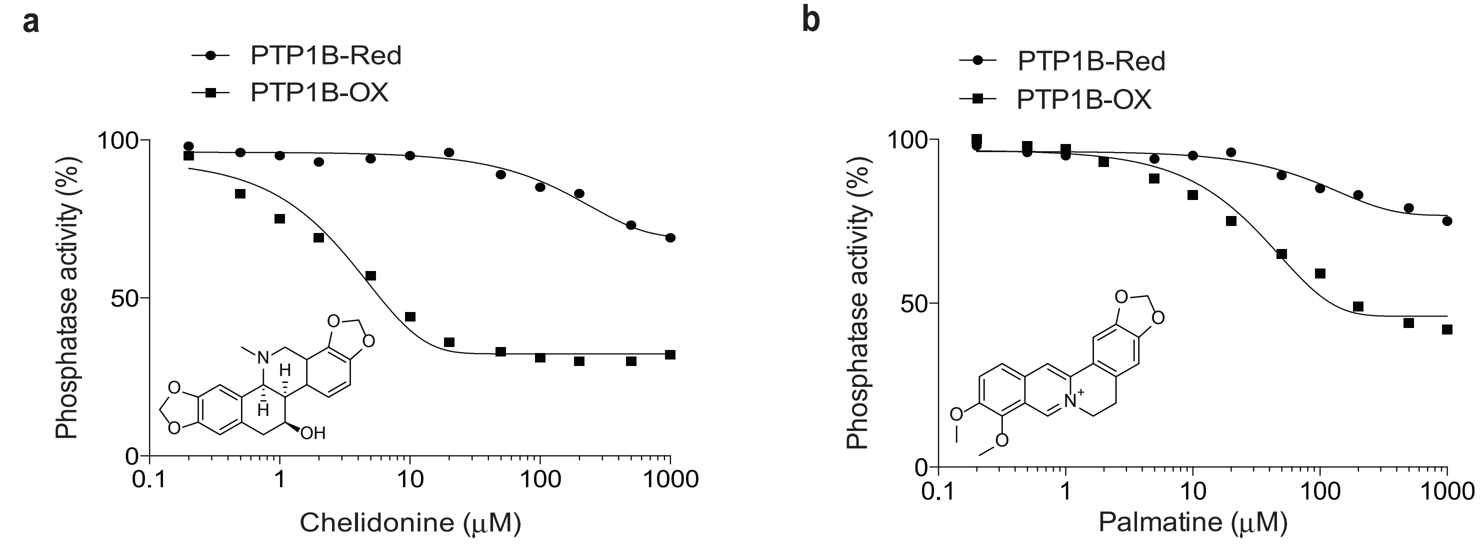

Inhibition of the reactivation of PTP1B-OX by analogs of sanguinarine.
A dose-response to test the effects of increasing concentrations of analogs of sanguinarine, chelidonine (a) and palmatine (b), tested against reduced (circle) and oxidized (square) forms of PTP1B, using DiFMUP as substrate. Individual data points represent the mean obtained from three separate experiments.

Supplementary Figure 8

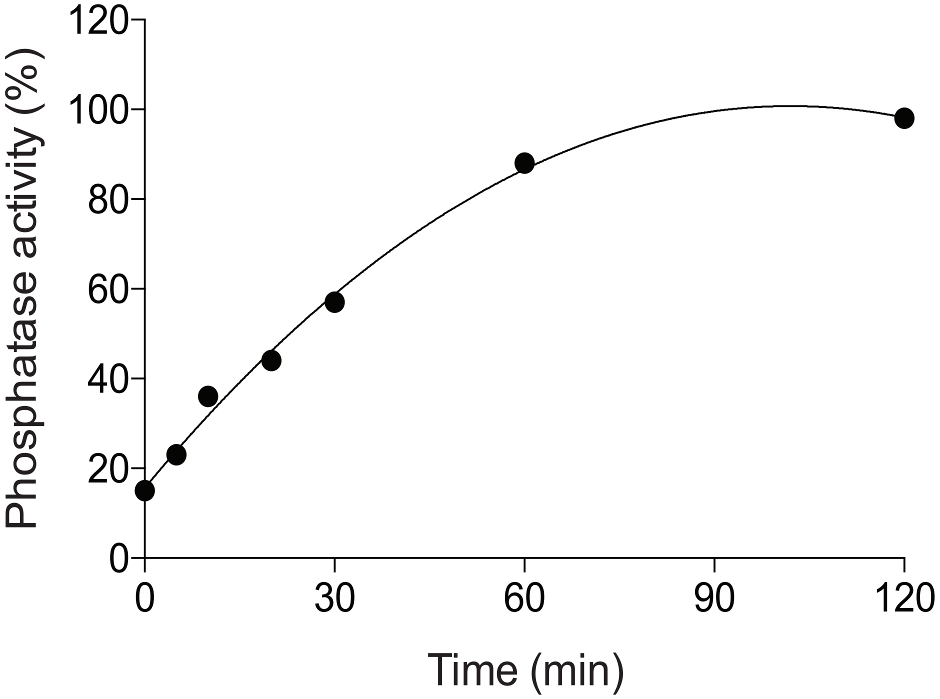

Reversibility of PTP1B-OX inhibition by chelerythrine.
PTP1B-OX (200 nM) was incubated with chelerythrine (5 μM) for 5 min, the complex was diluted 100-fold and the activity was monitored for 120 min continuously. Individual data points represent the mean obtained from three separate experiments.

Supplementary Figure 9

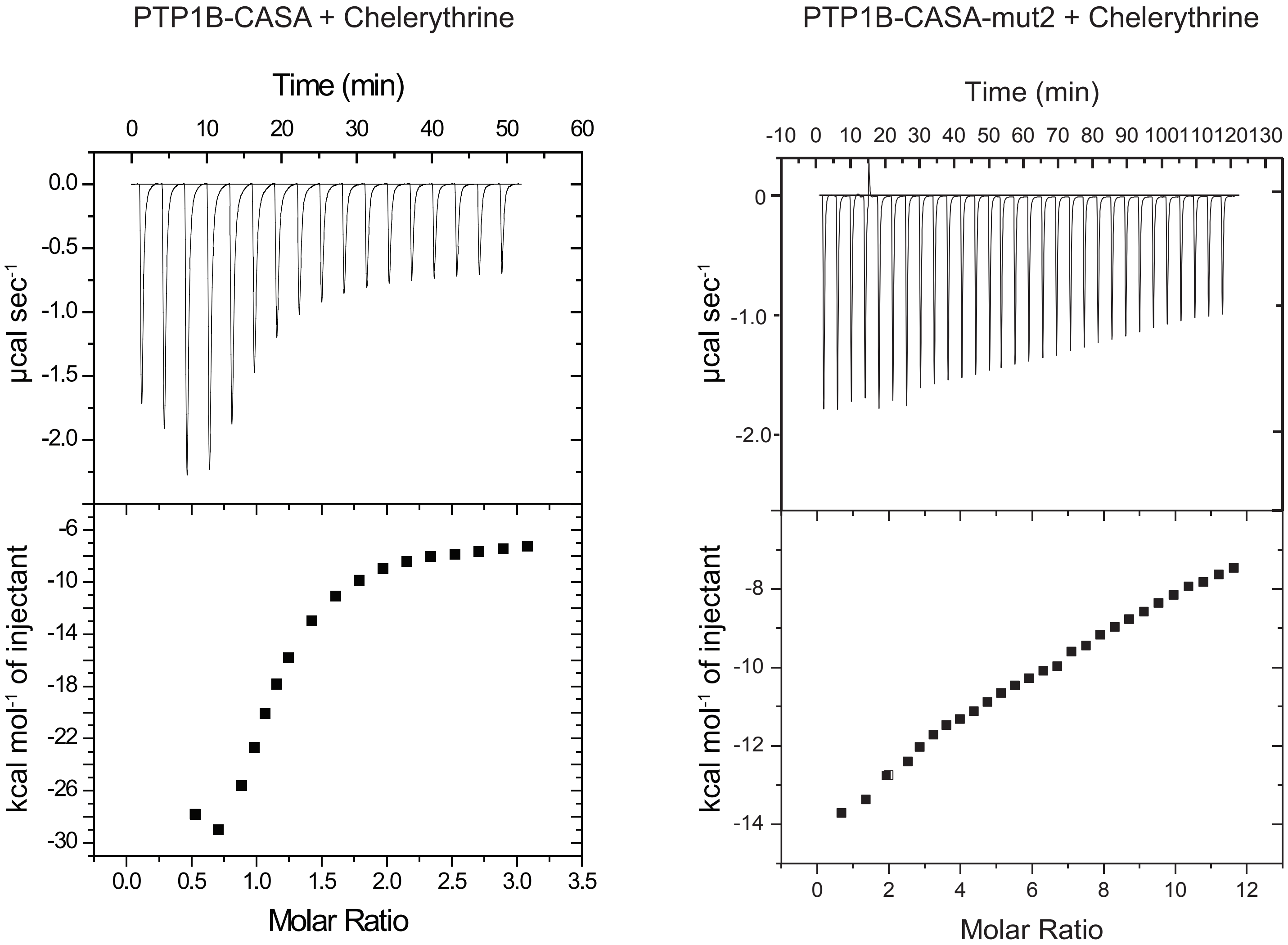

Isothermal titration calorimetry analysis of chelerythrine binding to PTP1B-CASA. Binding thermograms for PTP1B-CASA (left) and PTP1B-CASA-mut2 (right). The titration was set up for 60 min and saturation binding of chelerythrine to PTP1B-CASA was observed. The data was used to calculate a binding constant of 3 μM for the protein. In the case of PTP1B-CASA-mut2, titration was extended for 120 min to test if increasing the concentration of chelerythrine would facilitate binding of the compound to the mutant protein. No apparent binding of the compound to the mutant protein was observed; the binding could not be saturated and data could not be fit to obtain a binding constant. The data are representative of three independent experiments for PTP1B-CASA, in which the affinities were measured as 3.25 ± 0.36, 2.83 ± 0.15 and 3.44 ± 0.23 micromolar, and two independent experiments for PTP1B-CASA-mut2, in which we did not detect saturable binding.

Supplementary Figure 10

a

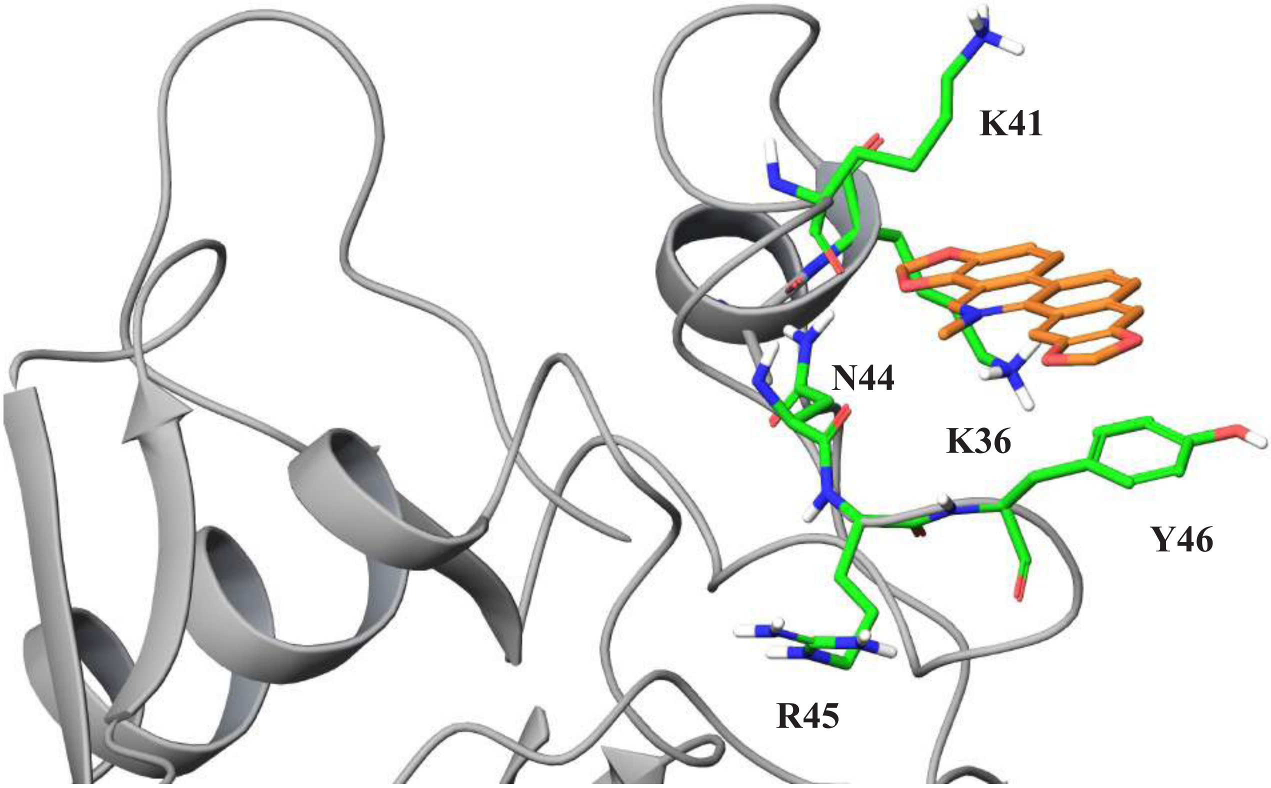

b

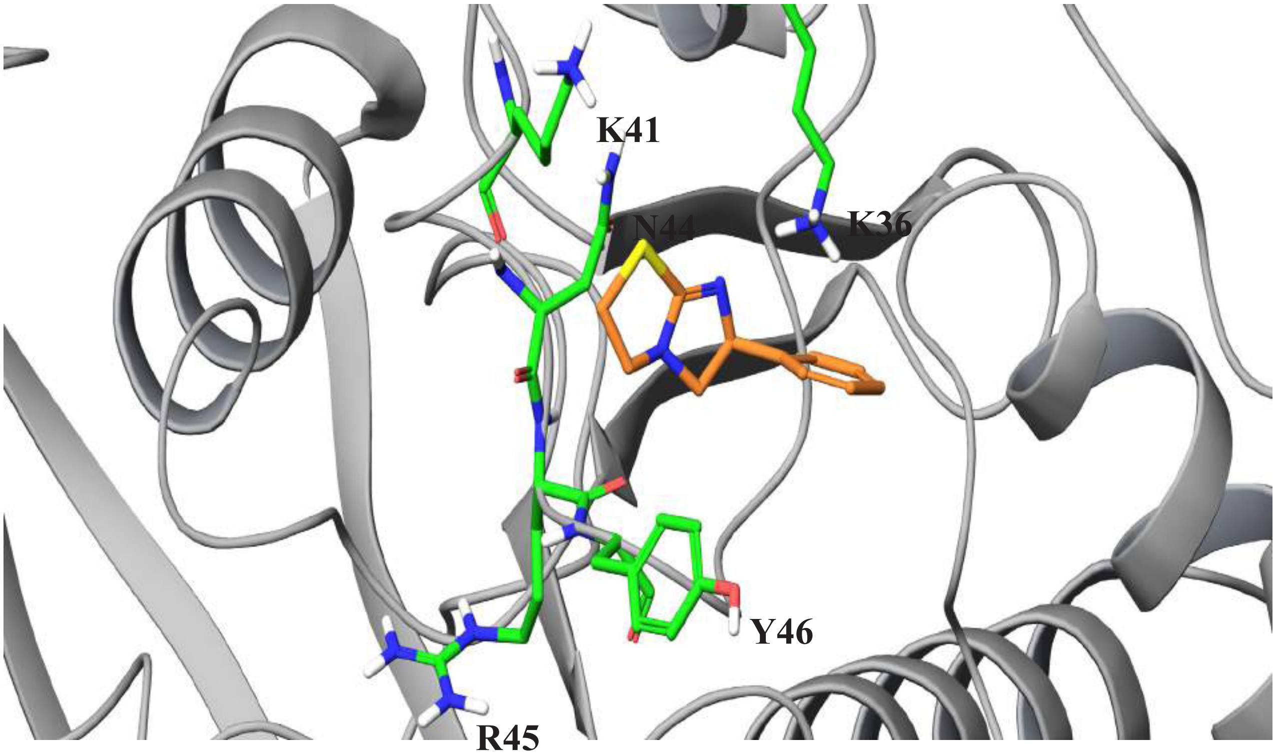

Molecular modeling of the interaction between PTP1B-OX and sanguinarine (a) and levamisole (b).
Residues critical for the interaction with each compound are highlighted.

Supplementary Figure 11

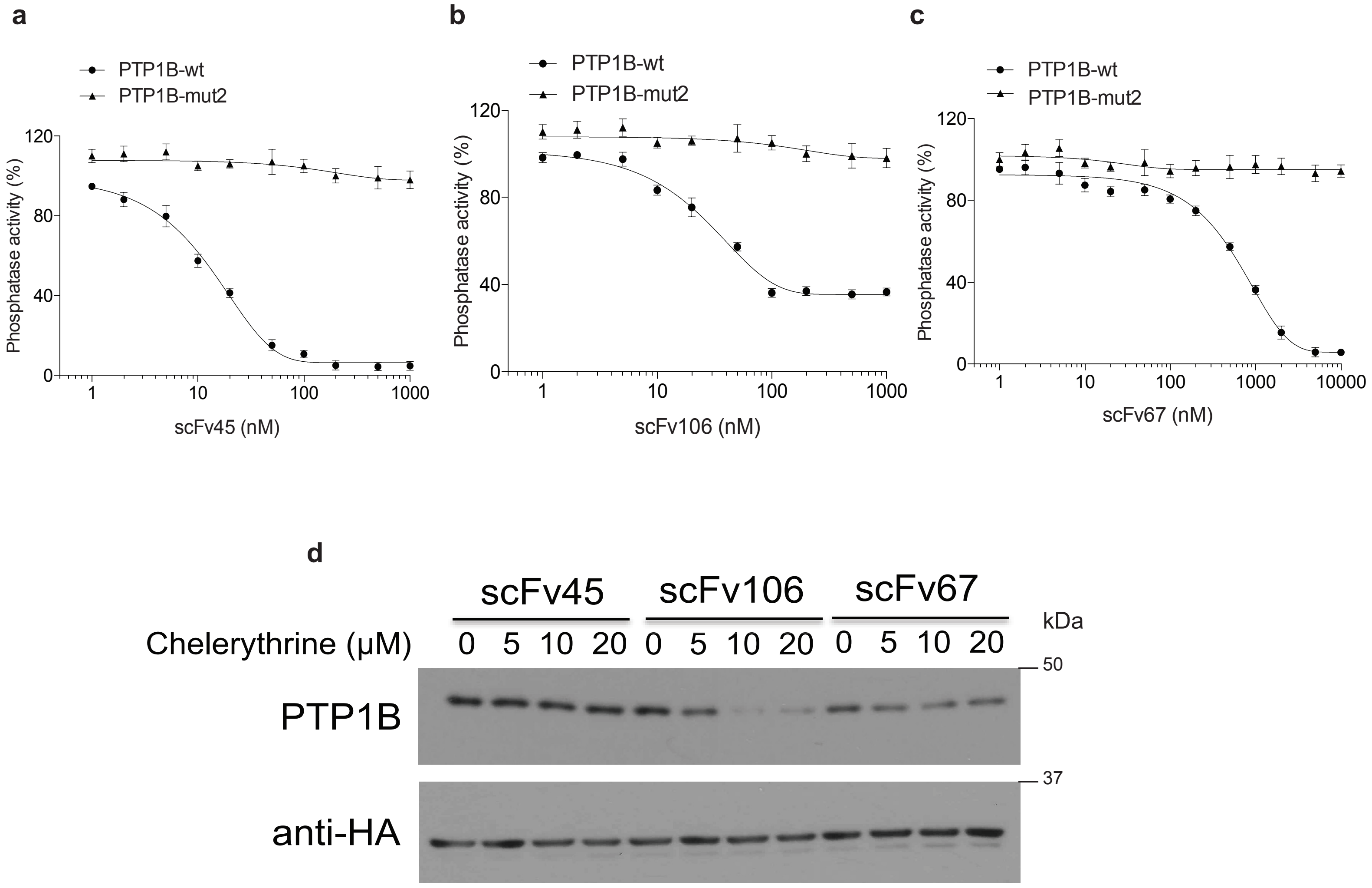

Displacement of intrabodies from PTP1B-OX by chelerythrine.
a-c) scFv45, scFv106 and scFv67 were tested against oxidized forms of PTP1B-wt and PTP1B-mut2. All three intrabodies inhibited PTP1B-wt (scFv45, Ki = 10 nM, scFv106, Ki = 100 nM (partial inhibitor) and scFv67, Ki = 1000 nM) but not PTP1B-mut2. Data represent mean ± s.e.m from three independent experiments. d) PTP1B-OX-scFv45, PTP1B-OX-scFv106 and PTP1B-OX-scFv67 complexes were titrated against chelerythrine (0 - 20 μM) and the extent to which the compound displaced the intrabodies bound to the protein was analyzed by immunoblotting for PTP1B. Data are representative of three independent experiments.

Supplementary Figure 12

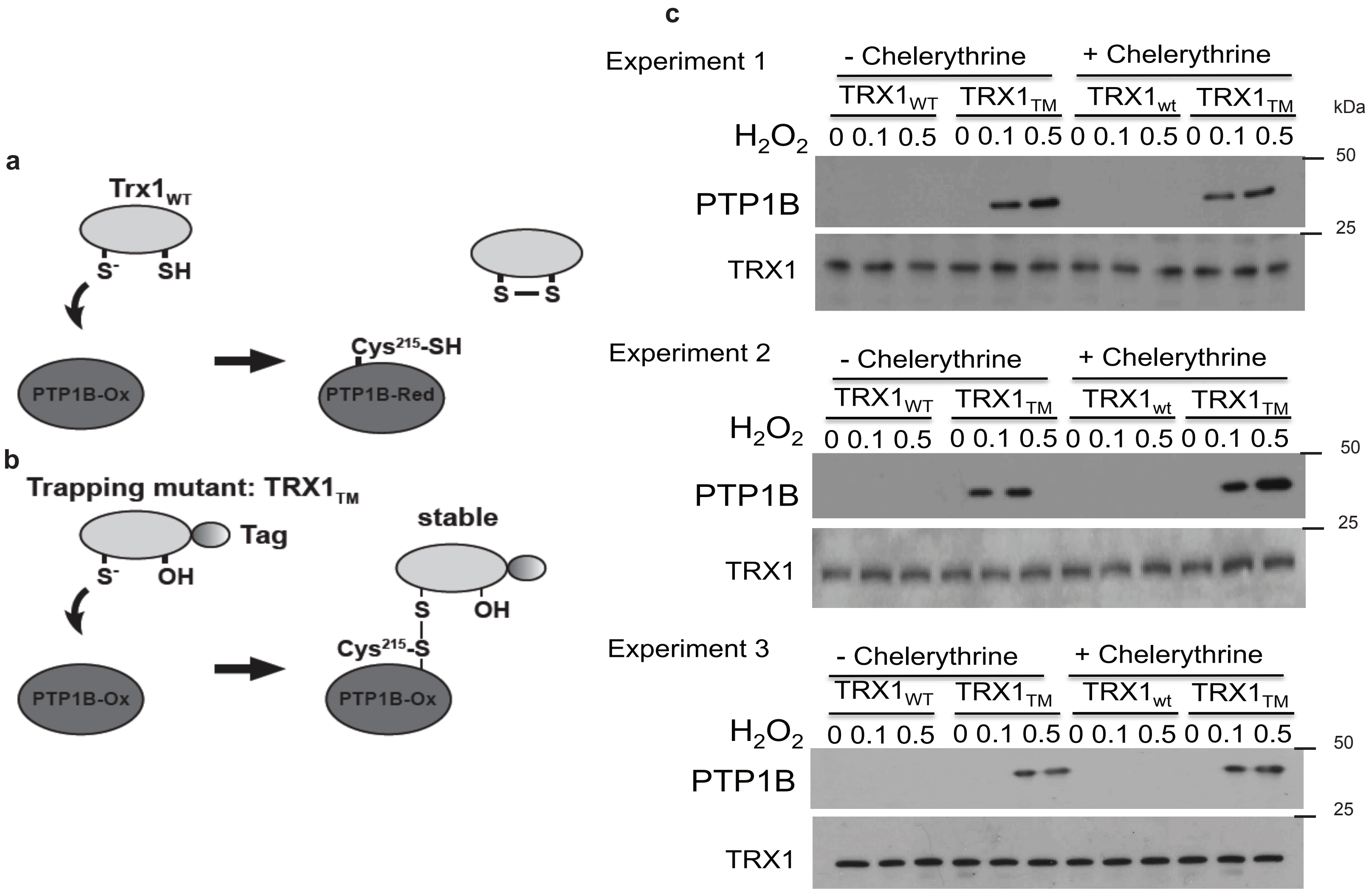

Thioredoxin was able to access the active site cysteine in PTP1B-OX in the absence and presence of chelerythrine. a) Cartoon to illustrate the mechanism by which TRX1-WT reduces and reactivates PTP1B-OX. b) Cartoon to illustrate the mechanism by which TRX1-TM (trapping mutant) forms a stable complex with PTP1B-OX. c) PTP1B was incubated with varying concentration of H_2O_2 (0-0.5 mM) and excess H_2O_2 was removed by running the protein through a desalting column. The protein was incubated with TRX1-WT or TRX1-TM in the absence and presence of chelerythrine (5 μM).
Data from three separate experiments are presented.

Supplementary Figure 13

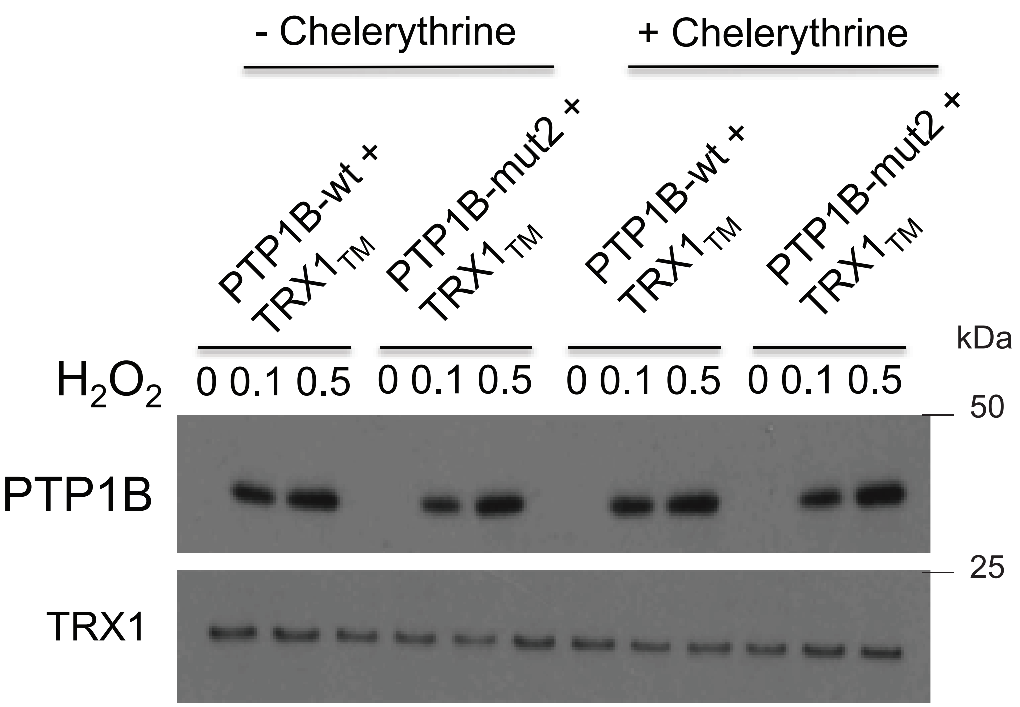

Thioredoxin did not differentiate between oxidized forms of PTP1B-wt and PTP1B-mut2. PTP1B-wt or PTP1B-mut2 was incubated with varying concentration of H_2O_2 (0-0.5 mM) and excess H_2O_2 was removed by running the protein through a desalting column. The protein was incubated with TRX1-TM in the absence and presence of chelerythrine (5 μM).
Data are representative of three independent experiments.

Supplementary Figure 14

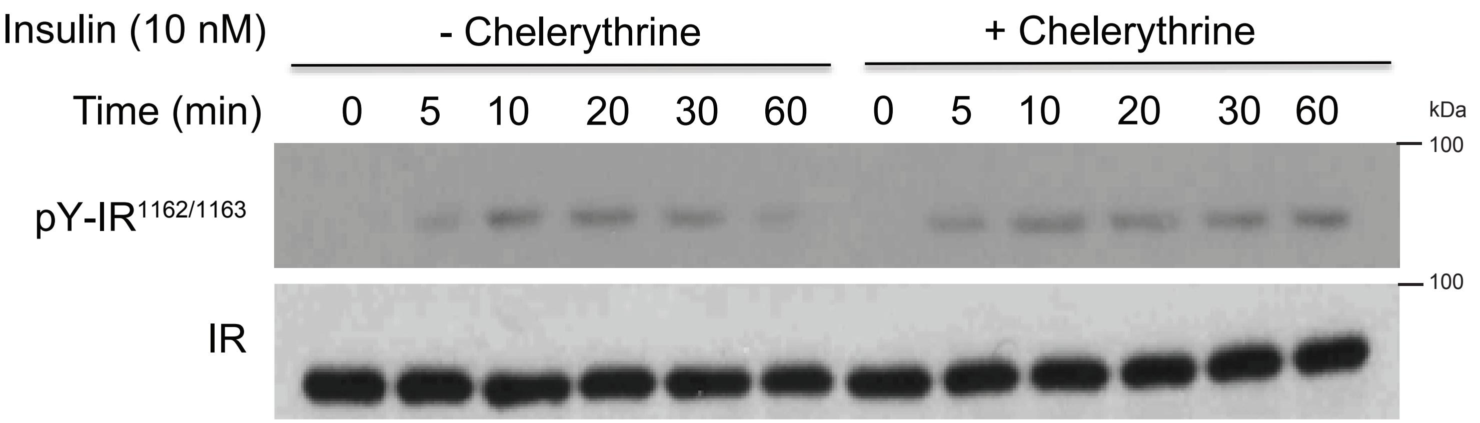

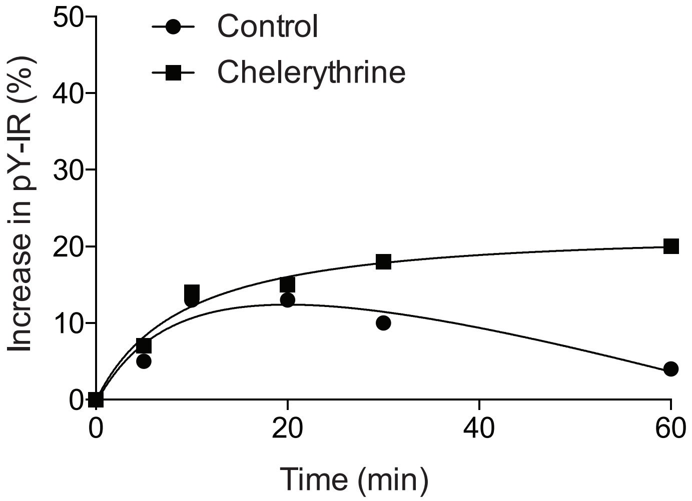

Insulin-induced signaling in 293T cells.

Cells, either untreated or treated with chelerythrine (2 μM) for 1 h, were stimulated with insulin (10 nM) for the indicated times, then lysed. Tyrosine phosphorylation of the beta-subunit of the insulin receptor was assessed by immunoblotting and quantified using Image J software (lower panel). Immunoblots are representative of three independent experiments.

Supplementary Figure 15

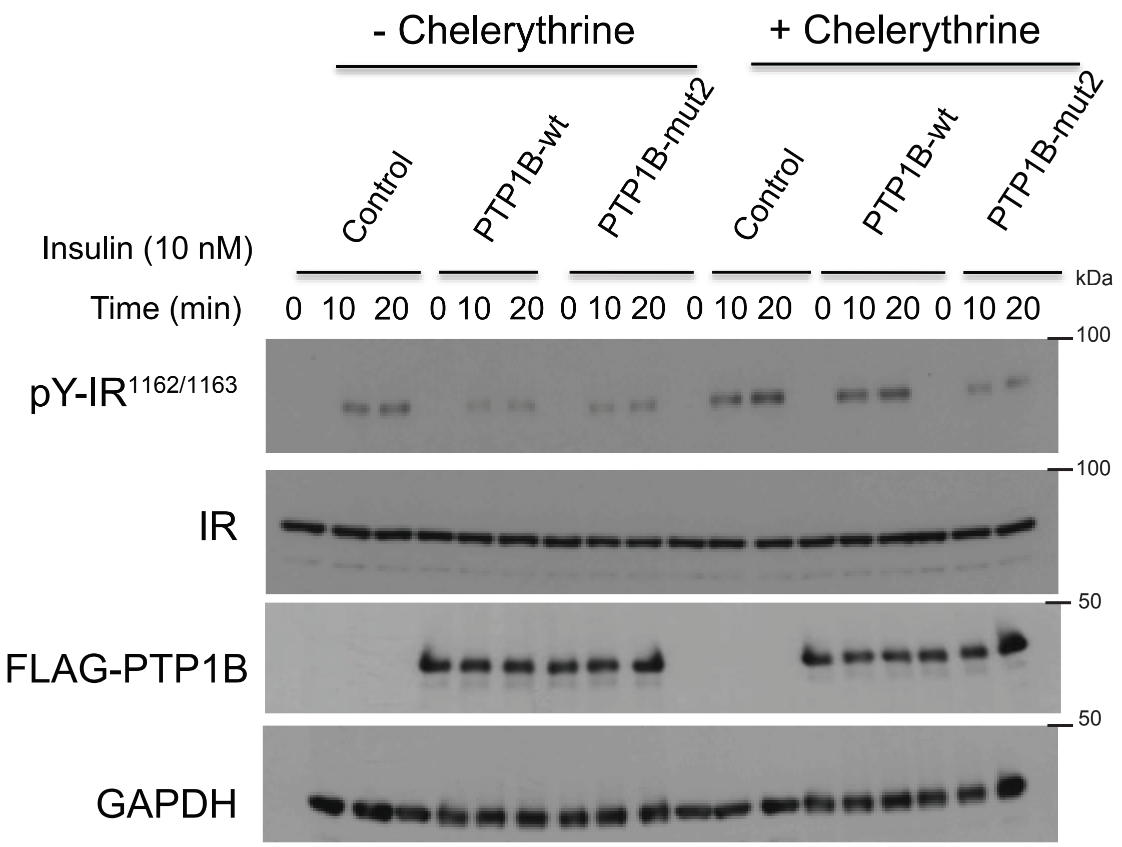

Chelerythrine inhibited PTP1B-OX and not PTP1B-mut2-OX. Cells transfected with vector, PTP1B-wt or PTP1B-mut2, either untreated or treated with chelerythrine (2 μM) for 1h, were stimulated with insulin (10 nM) for the indicated times. Tyrosine phosphorylation of the beta-subunit of the insulin receptor was then assessed by immunoblotting. Data are representative of three independent experiments.

Supplementary Figure 16

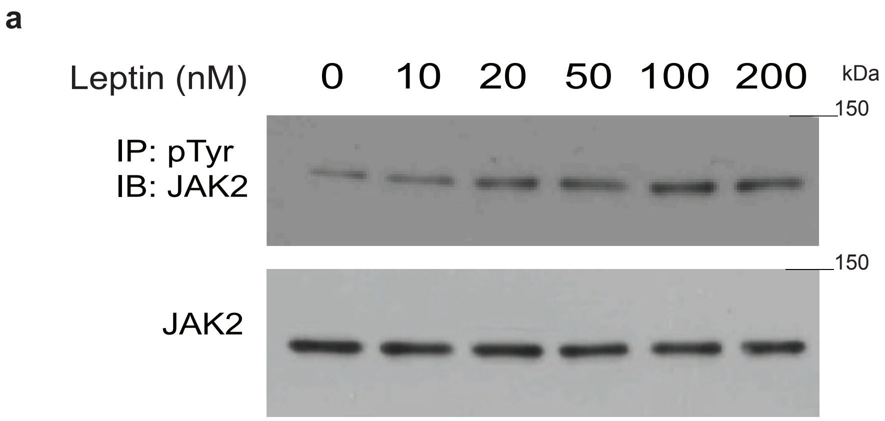

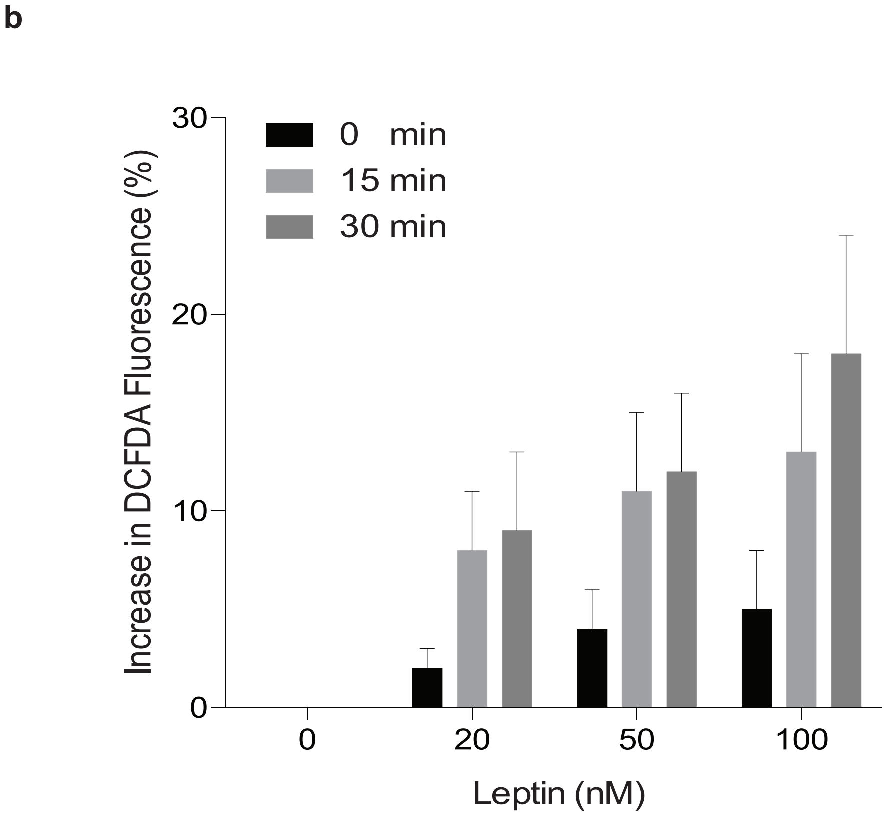

Leptin-induced signaling in hepatic stellate cells. a) Hepatic stellate cells were stimulated with varying concentration of leptin (0-200 nM), cells were lysed and pTyr proteins were immunprecipitated, then immunoblotted with anti-JAK2 antibody. Data are representative of at least three independent experiments. b) Hepatic stellate cells were stimulated with varying concentration of leptin (0-100 nM), and leptin-induced ROS production was monitored using 2,7-dichloroflourescein diacetate. Data represent the mean ± s.e.m. from at least three independent experiments.

Supplementary Figure 17

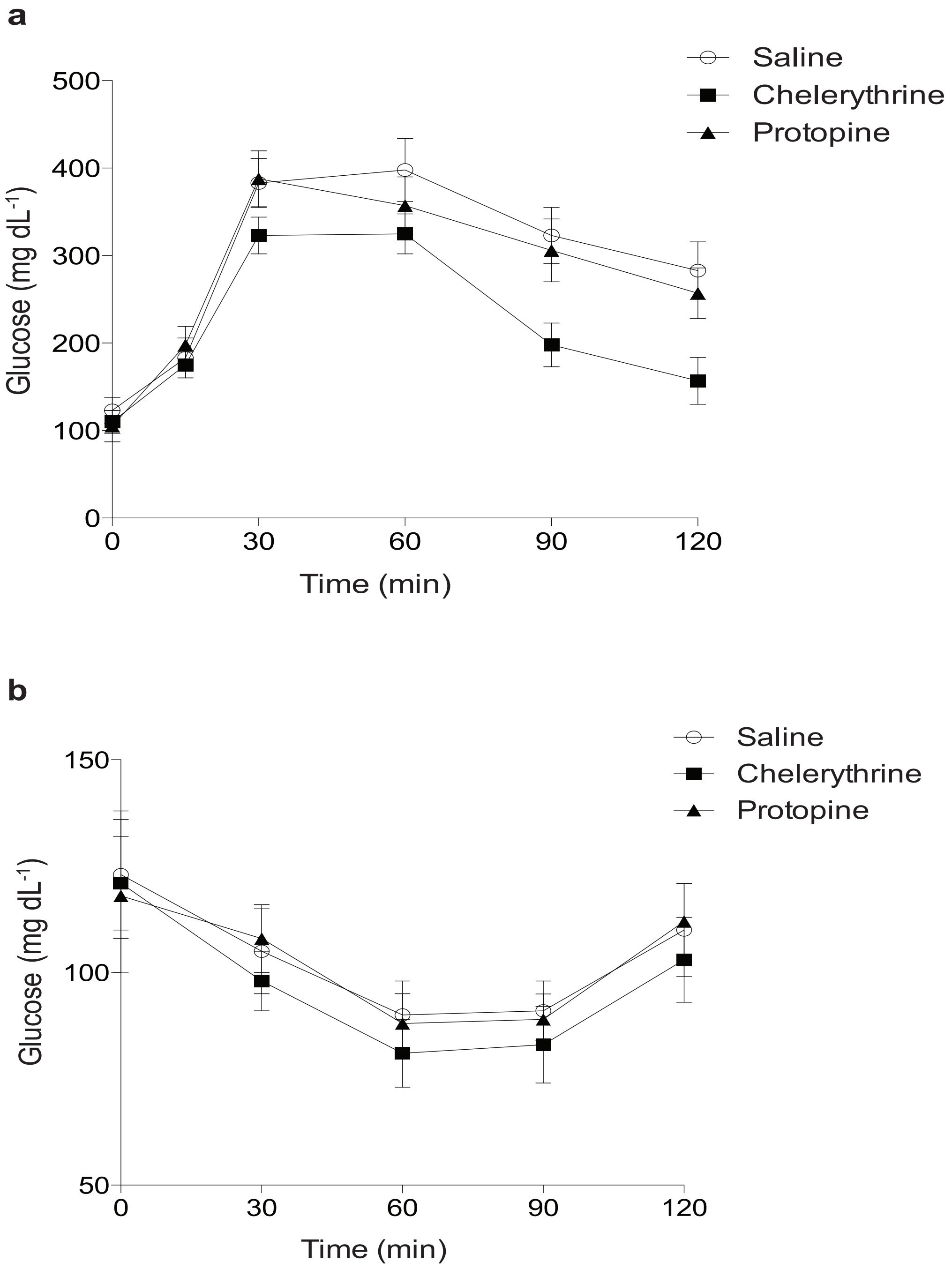

Protopine unlike chelerythrine did not improve glucose homeostasis in HFD fed mice. a) HFD fed 14-week-old male mice treated with saline, chelerythrine or protopine, were administered D-glucose (2 mg g^{-1} BW), and blood glucose was monitored (n = 10 in each group). b) HFD fed 14-week-old male mice treated with saline, chelerythrine or protopine, were administered insulin (0.75 mU g^{-1} BW), and blood glucose was monitored (n = 10 in each group). Data represent the mean ± s.e.m.

Supplementary Figure 18

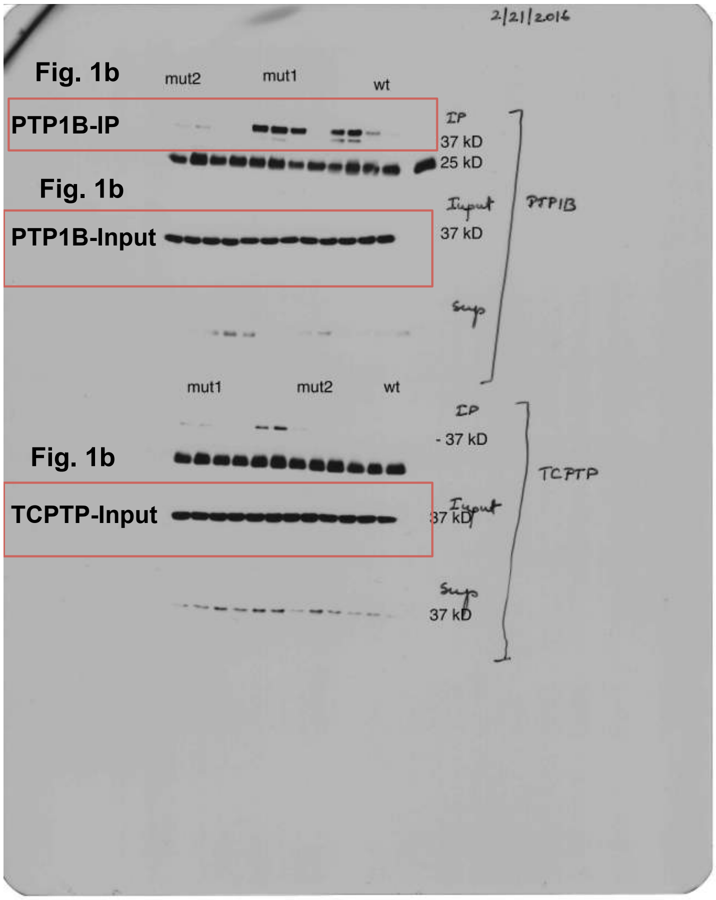

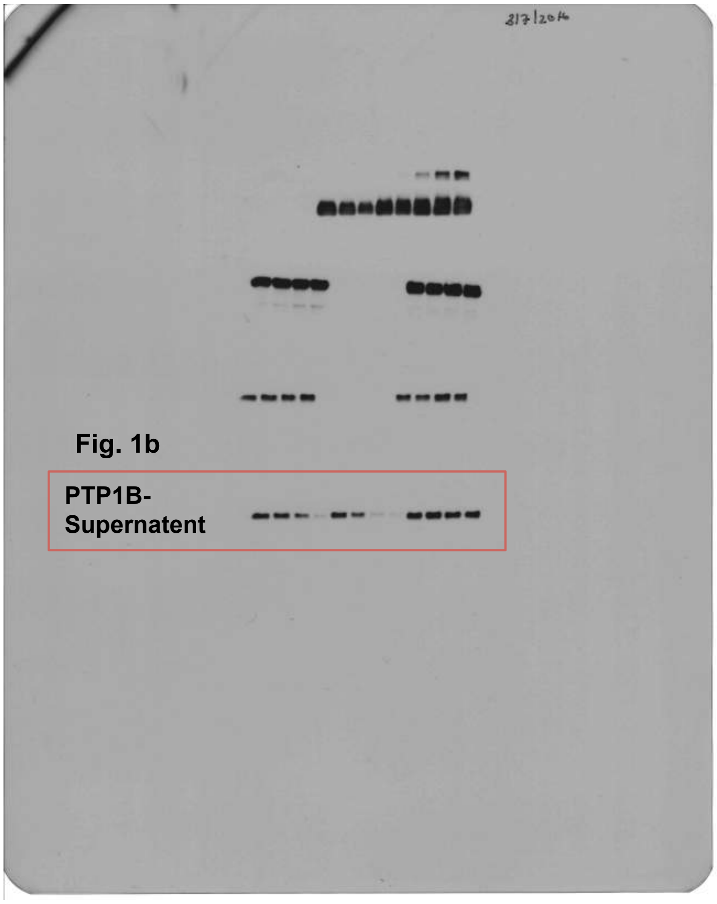

Uncropped immunoblot images from Figure 1. Shown are uncropped immunoblots presented in Fig. 1b. Images are labeled corresponding to their respective data from the manuscript, each individually denoted by a red box. Data are representative of three independent experiments.

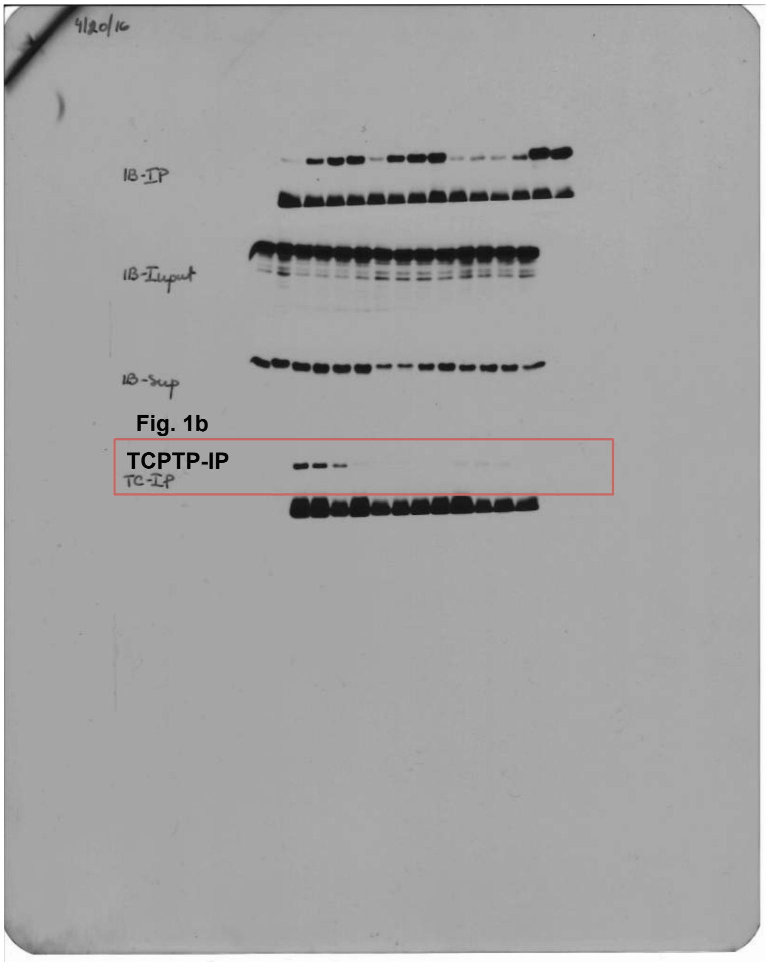

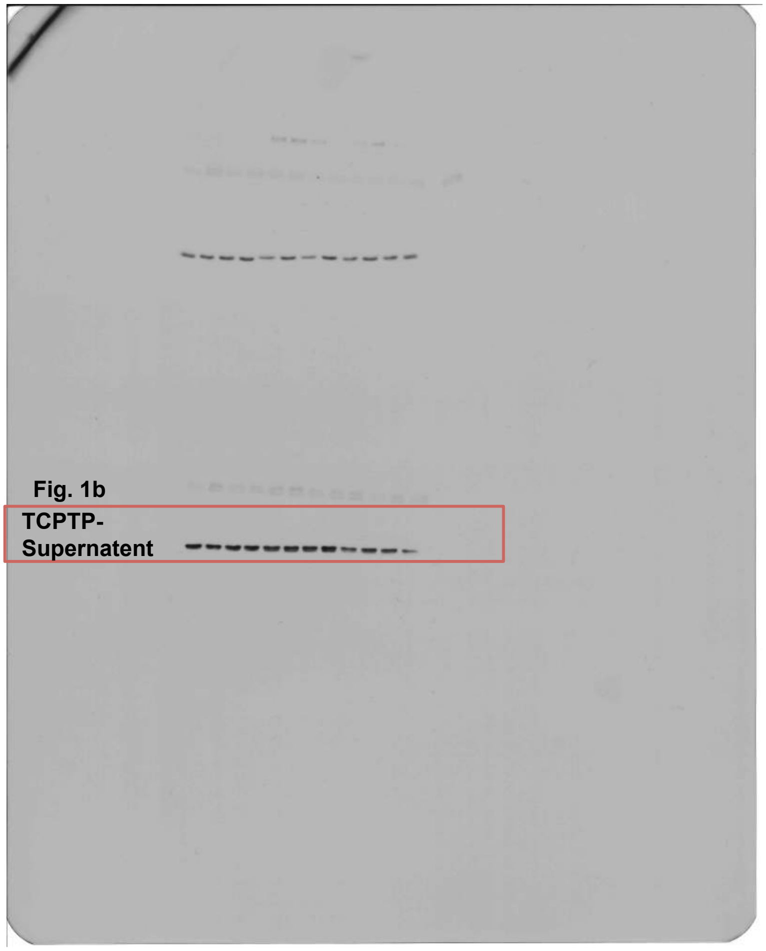

Uncropped immunoblot images from Figure 1. Shown are uncropped immunoblots presented in Fig. 1b. Images are labeled corresponding to their respective data from the manuscript, each individually denoted by a red box. Data are representative of three independent experiments.

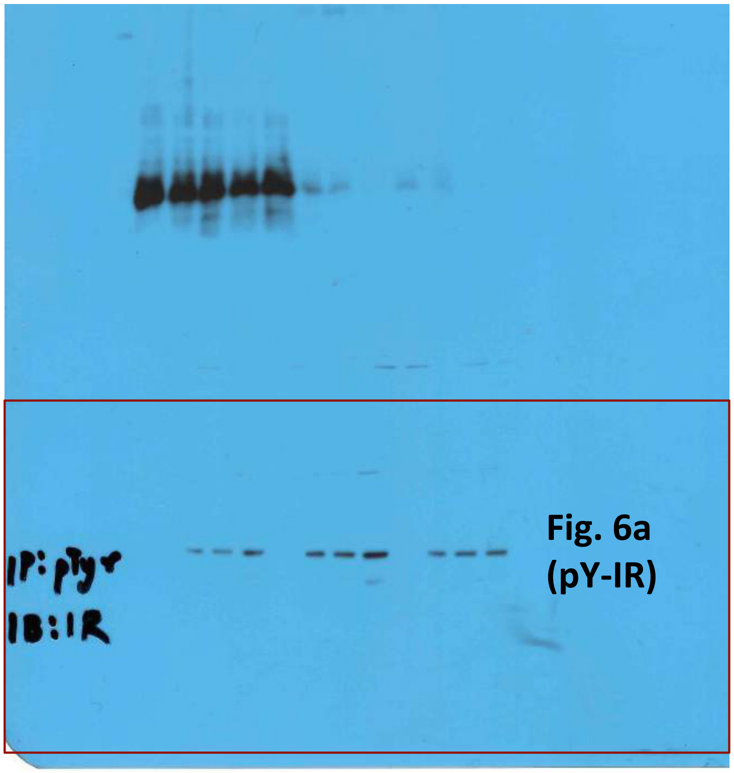

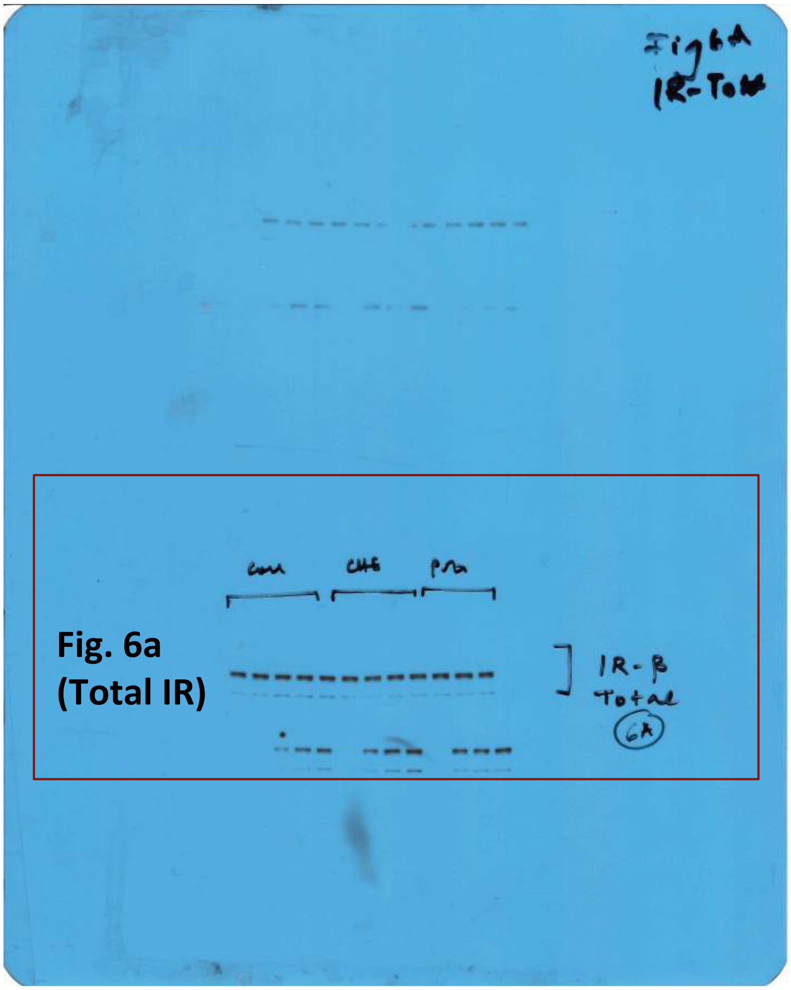

Uncropped immunoblot images from Figure 6. Shown are uncropped immunoblots presented in Fig. 6a. Images are labeled corresponding to their respective data from the manuscript, each individually denoted by a red box. Data are representative of three independent experiments.

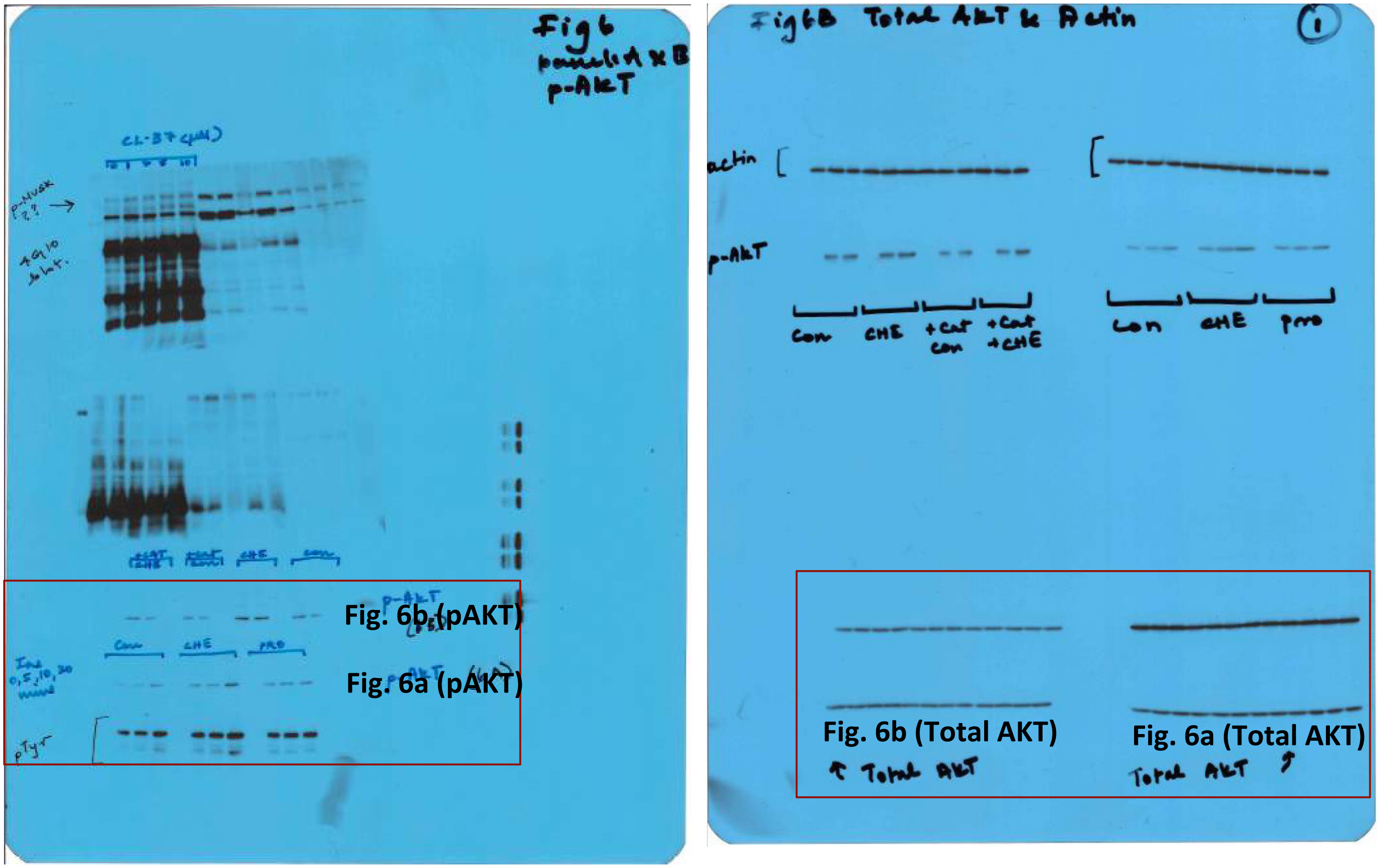

Uncropped immunoblot images from Figure 6. Shown are uncropped immunoblots presented in Fig. 6a & 6b. Images are labeled corresponding to their respective data from the manuscript, each individually denoted by a red box. Data are representative of three independent experiments.

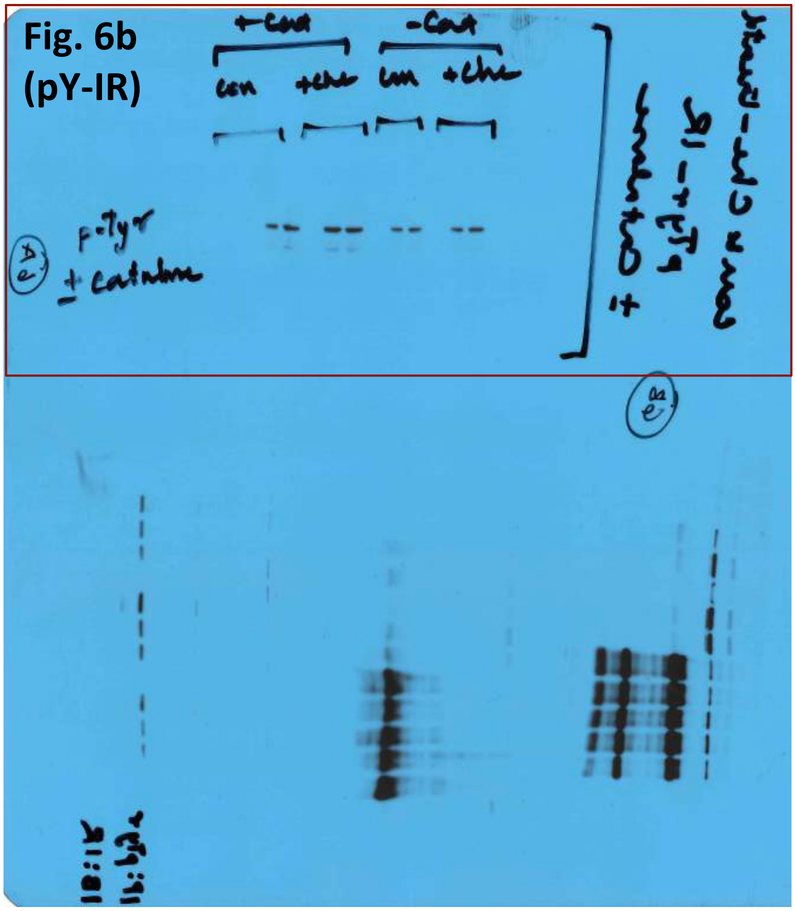

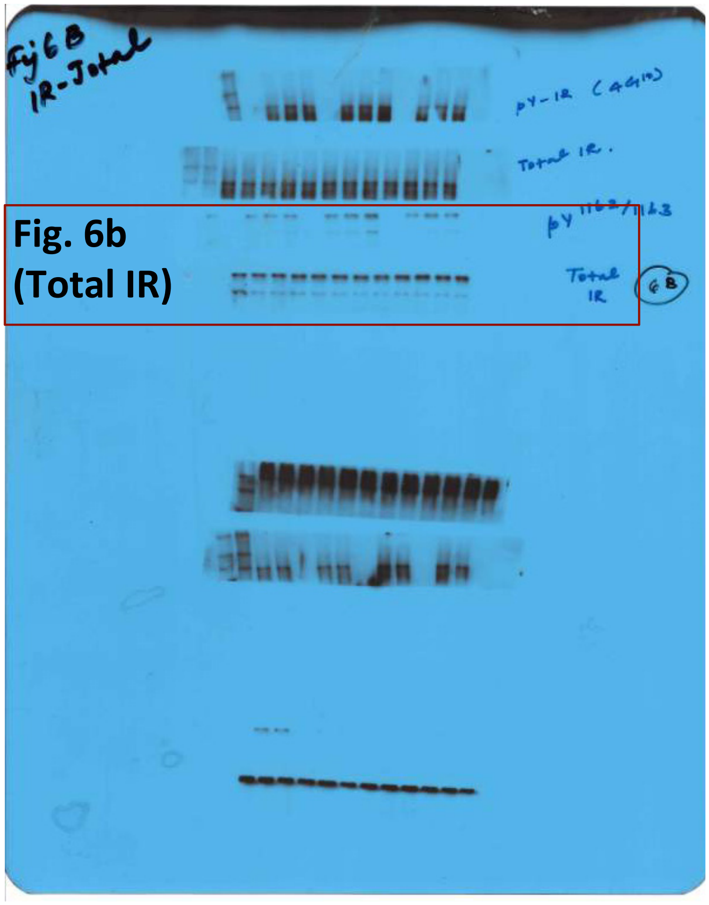

Uncropped immunoblot images from Figure 6. Shown are uncropped immunoblots presented in Fig. 6b. Images are labeled corresponding to their respective data from the manuscript, each individually denoted by a red box. Data are representative of three independent experiments.

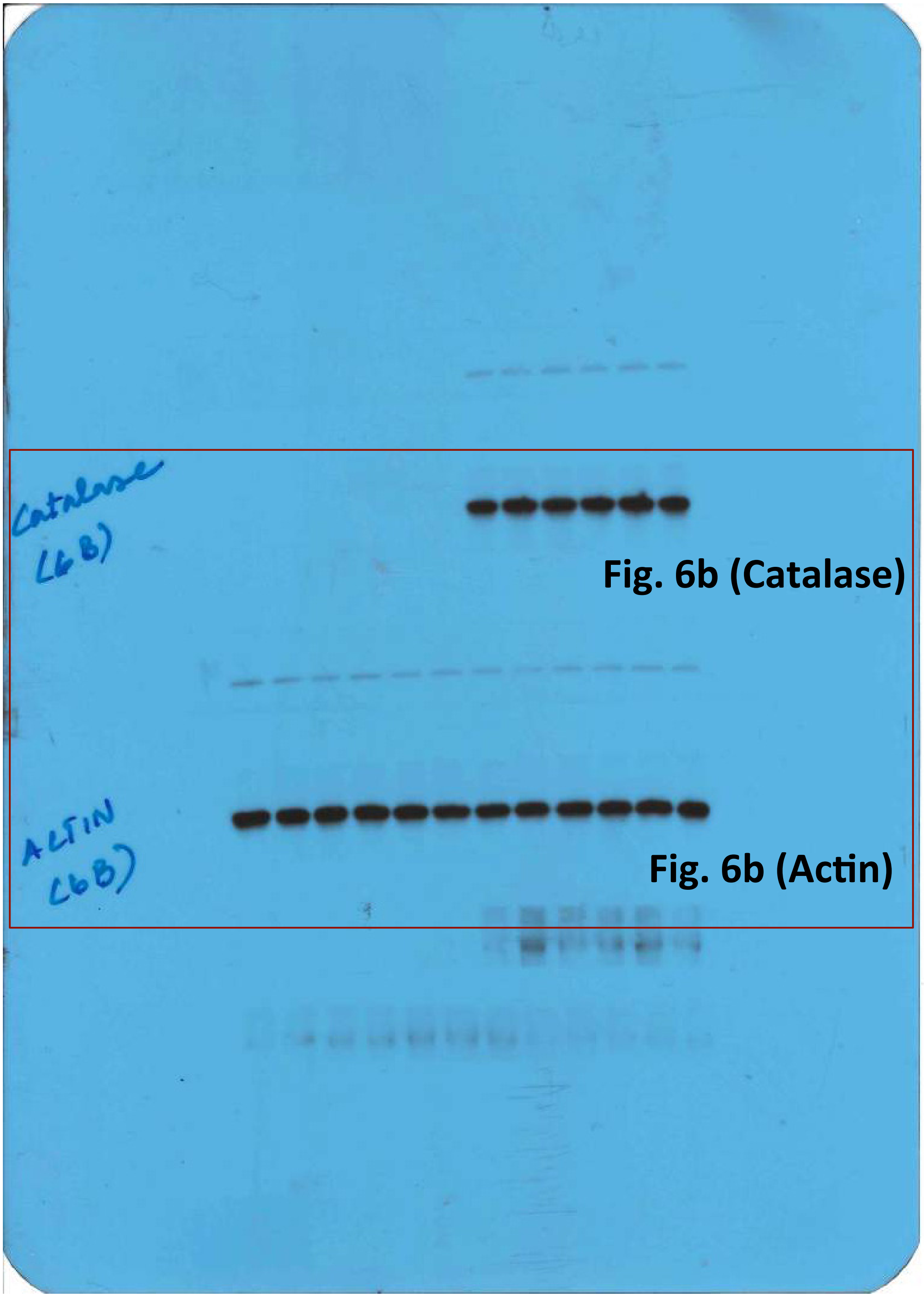

Uncropped immunoblot images from Figure 6. Shown are uncropped immunoblots presented in Fig. 6b. Images are labeled corresponding to their respective data from the manuscript, each individually denoted by a red box. Data are representative of three independent experiments.

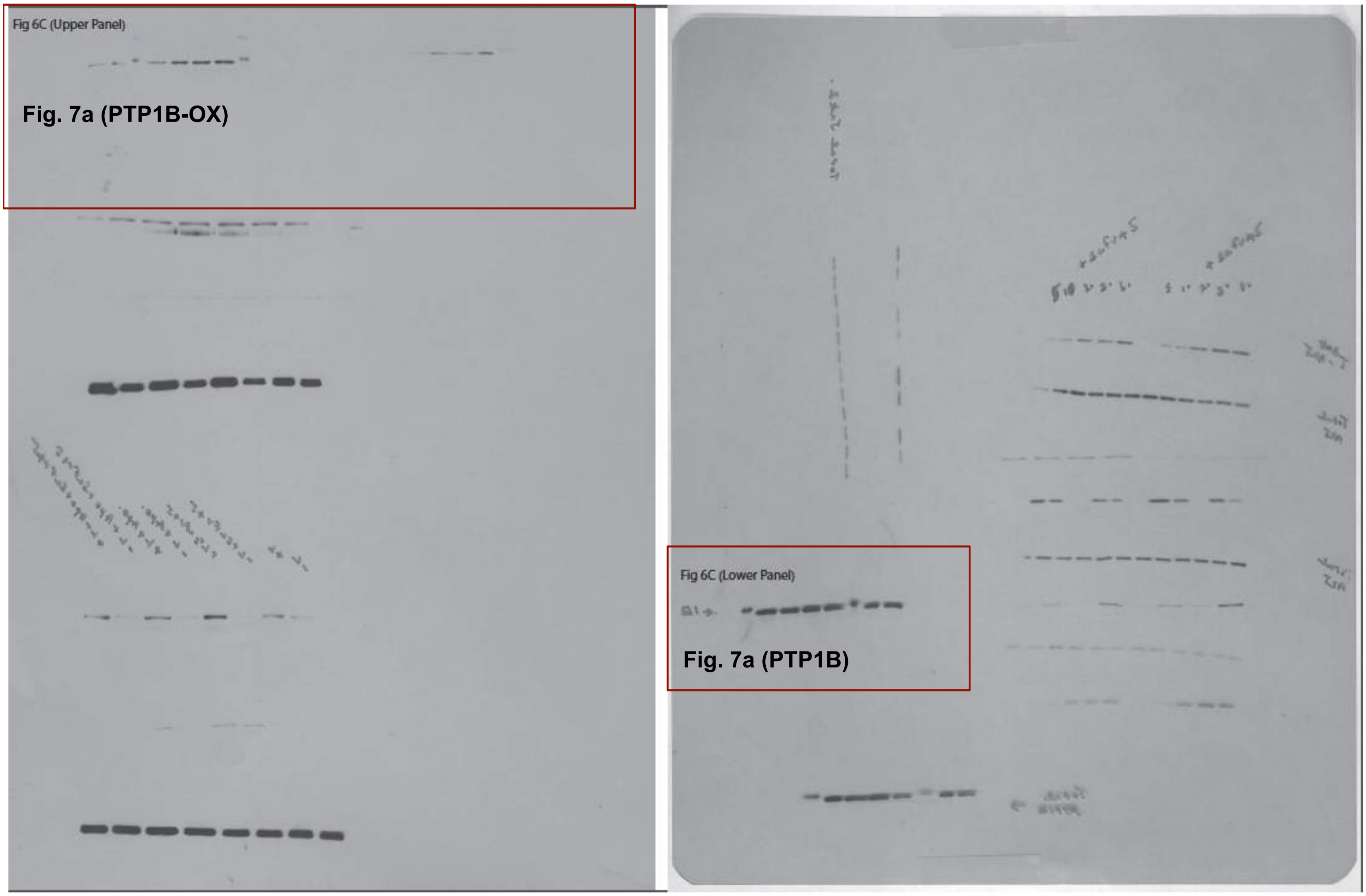

Uncropped immunoblot images from Figure 7. Shown are uncropped immunoblots presented in Fig. 7a. Images are labeled corresponding to their respective data from the manuscript, each individually denoted by a red box. Data are representative of three independent experiments.

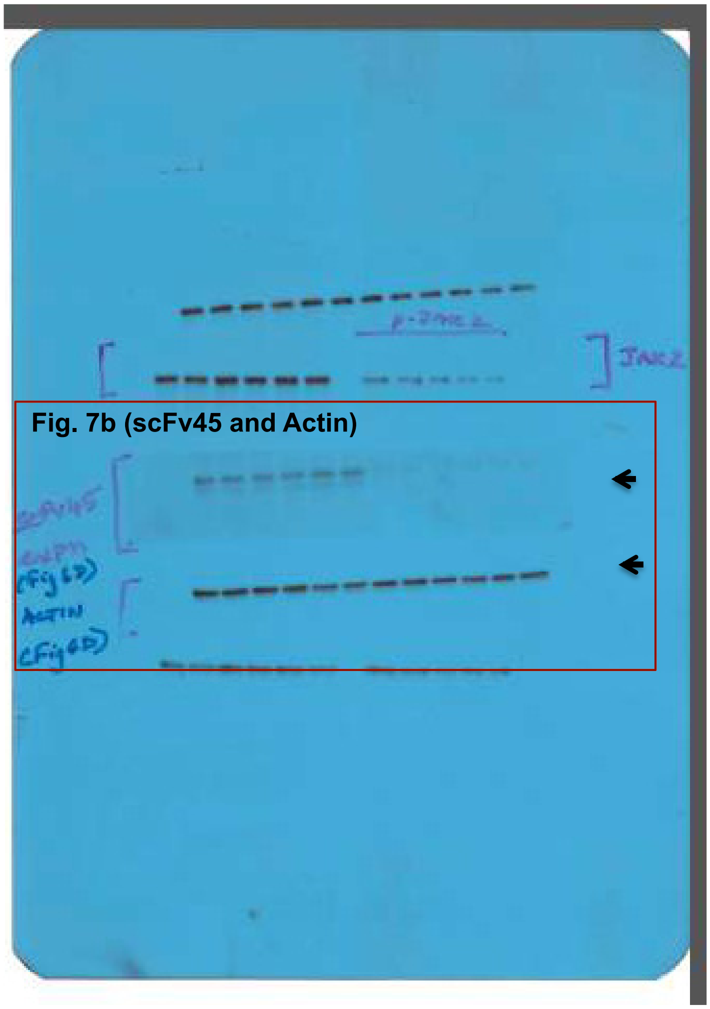

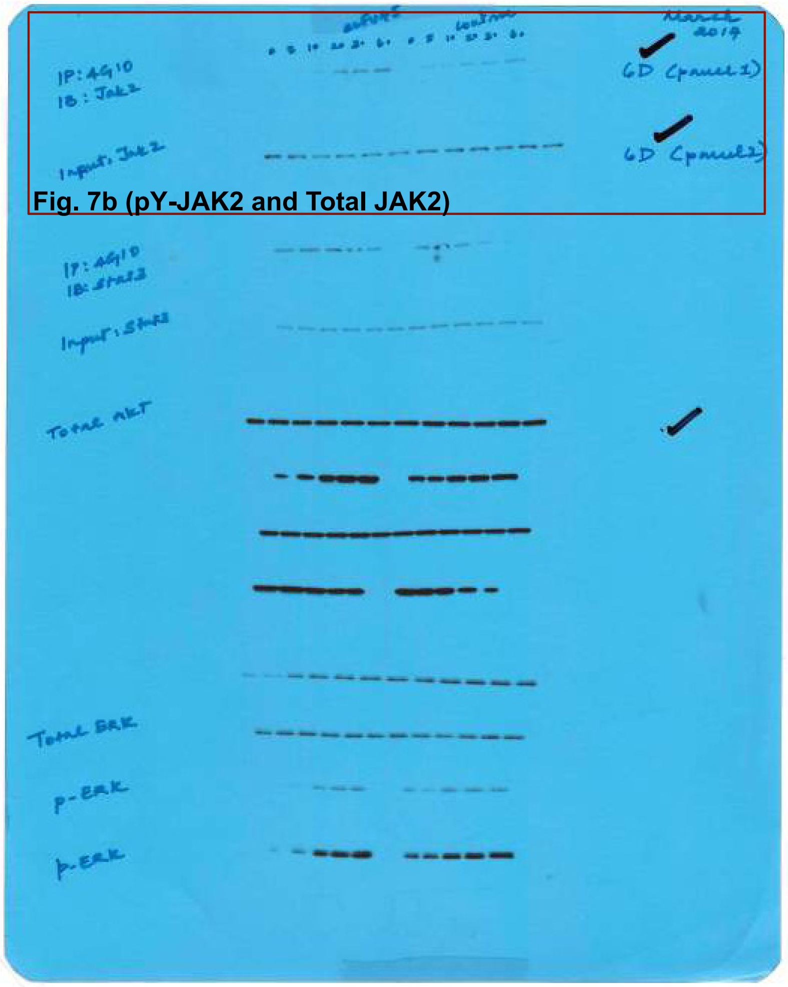

Uncropped immunoblot images from Figure 7. Shown are uncropped immunoblots presented in Fig. 7b. Images are labeled corresponding to their respective data from the manuscript, each individually denoted by a red box. Data are representative of three independent experiments.

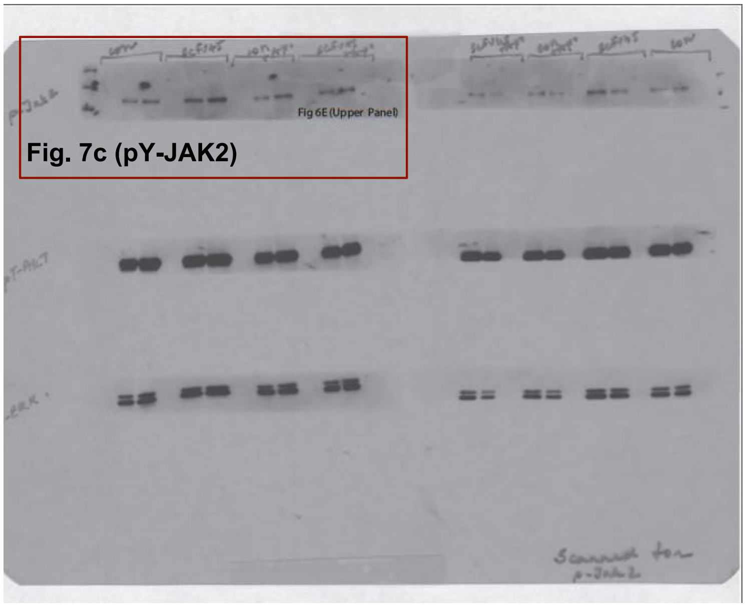

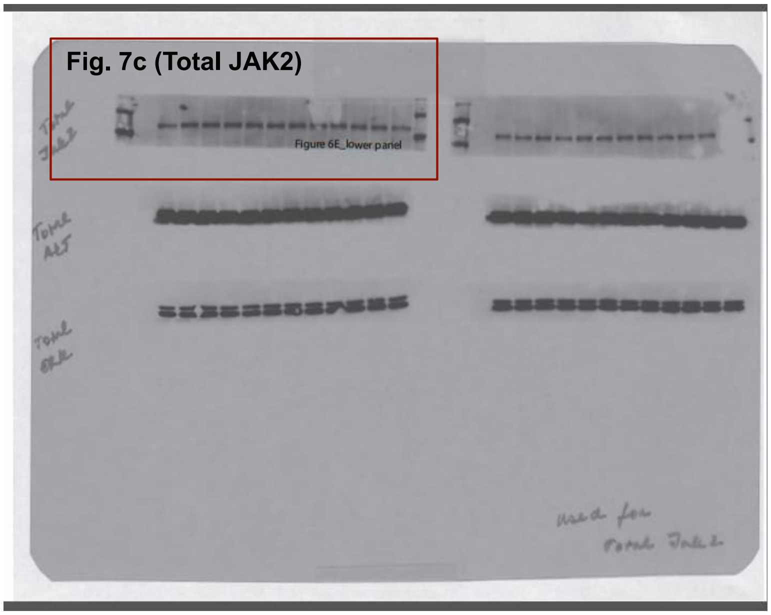

Uncropped immunoblot images from Figure 7. Shown are uncropped immunoblots presented in Fig. 7c. Images are labeled corresponding to their respective data from the manuscript, each individually denoted by a red box. Data are representative of three independent experiments.

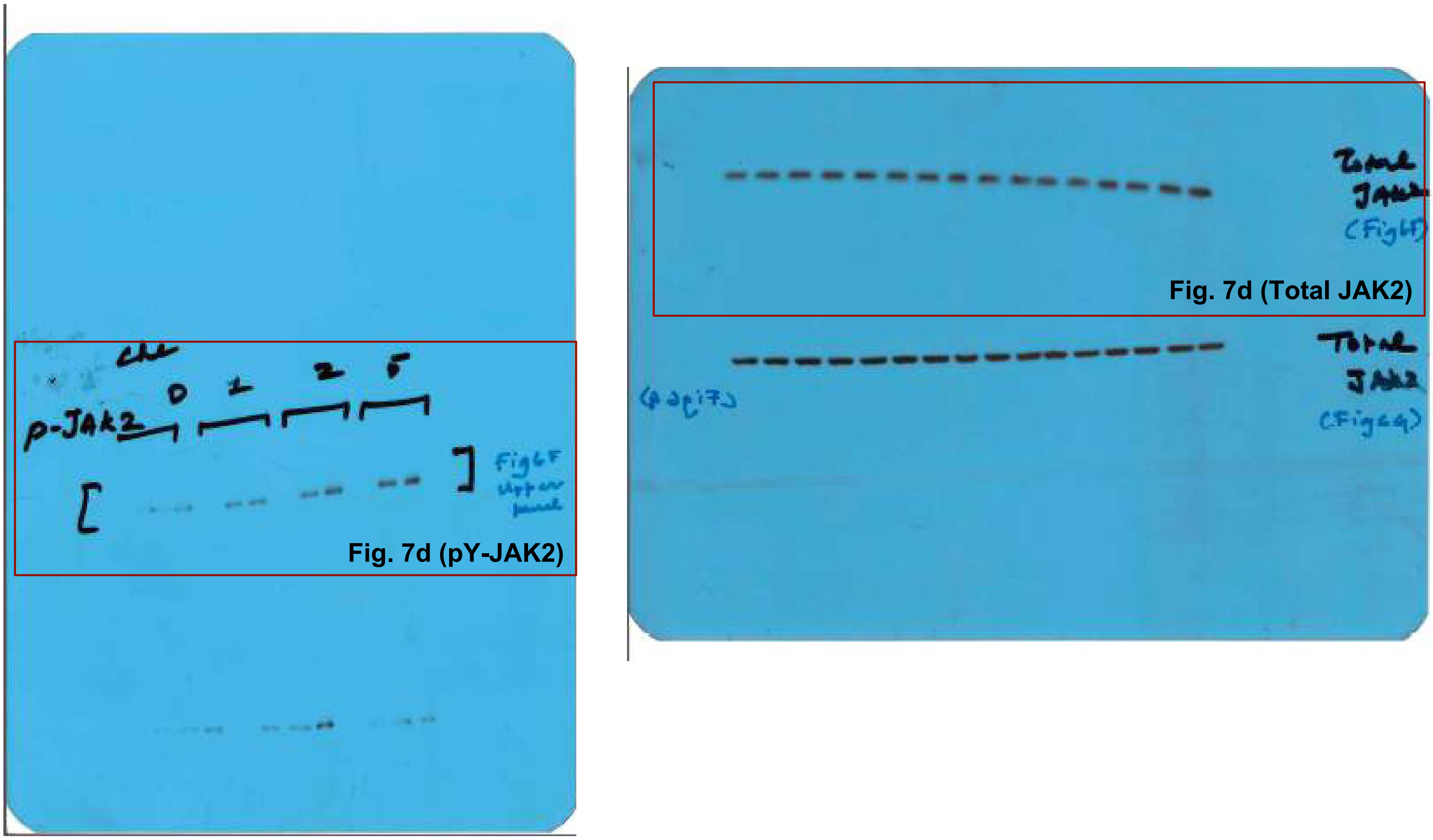

Uncropped immunoblot images from Figure 7. Shown are uncropped immunoblots presented in Fig. 7d. Images are labeled corresponding to their respective data from the manuscript, each individually denoted by a red box. Data are representative of three independent experiments.

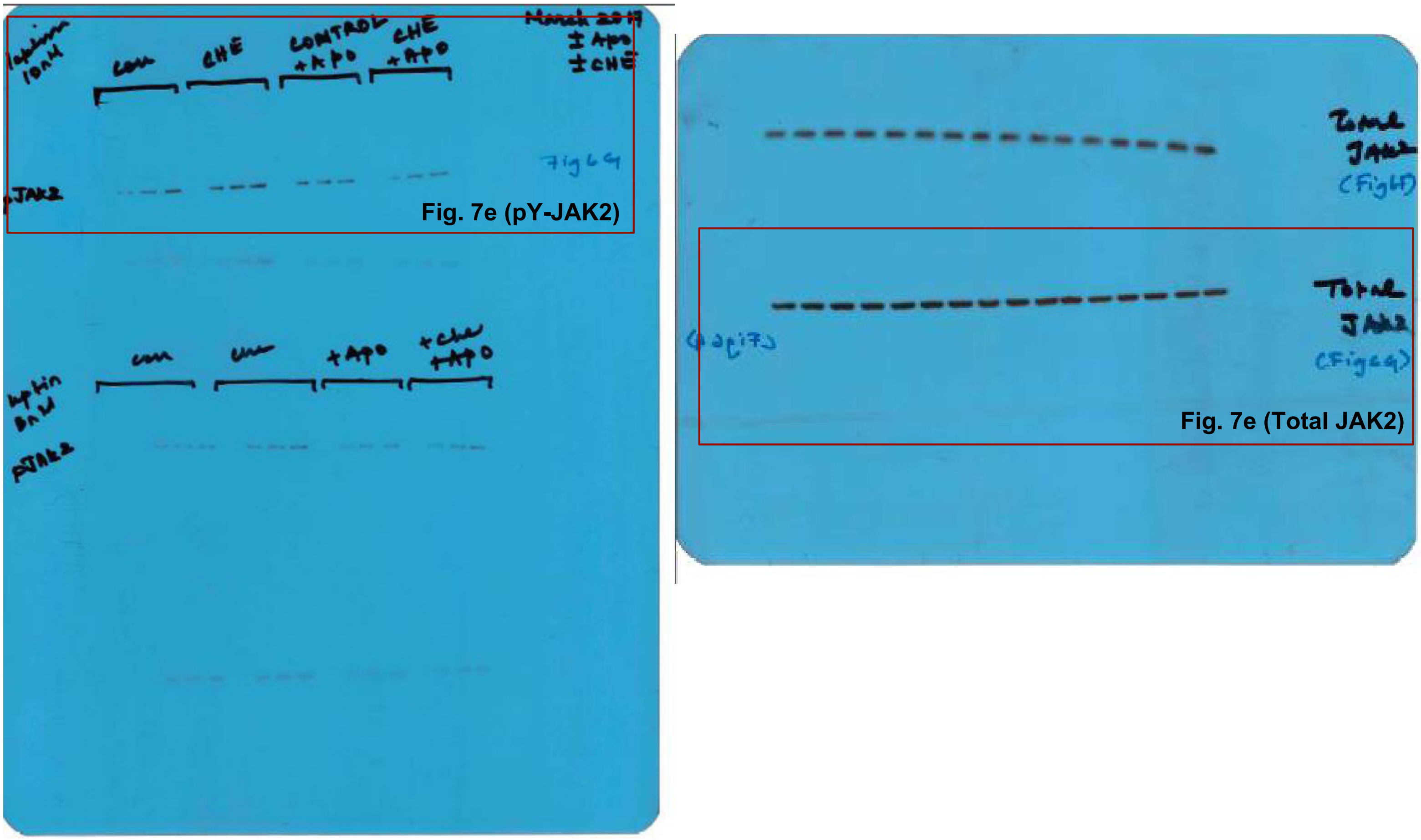

Uncropped immunoblot images from Figure 7. Shown are uncropped immunoblots presented in Fig. 7e. Images are labeled corresponding to their respective data from the manuscript, each individually denoted by a red box. Data are representative of three independent experiments.

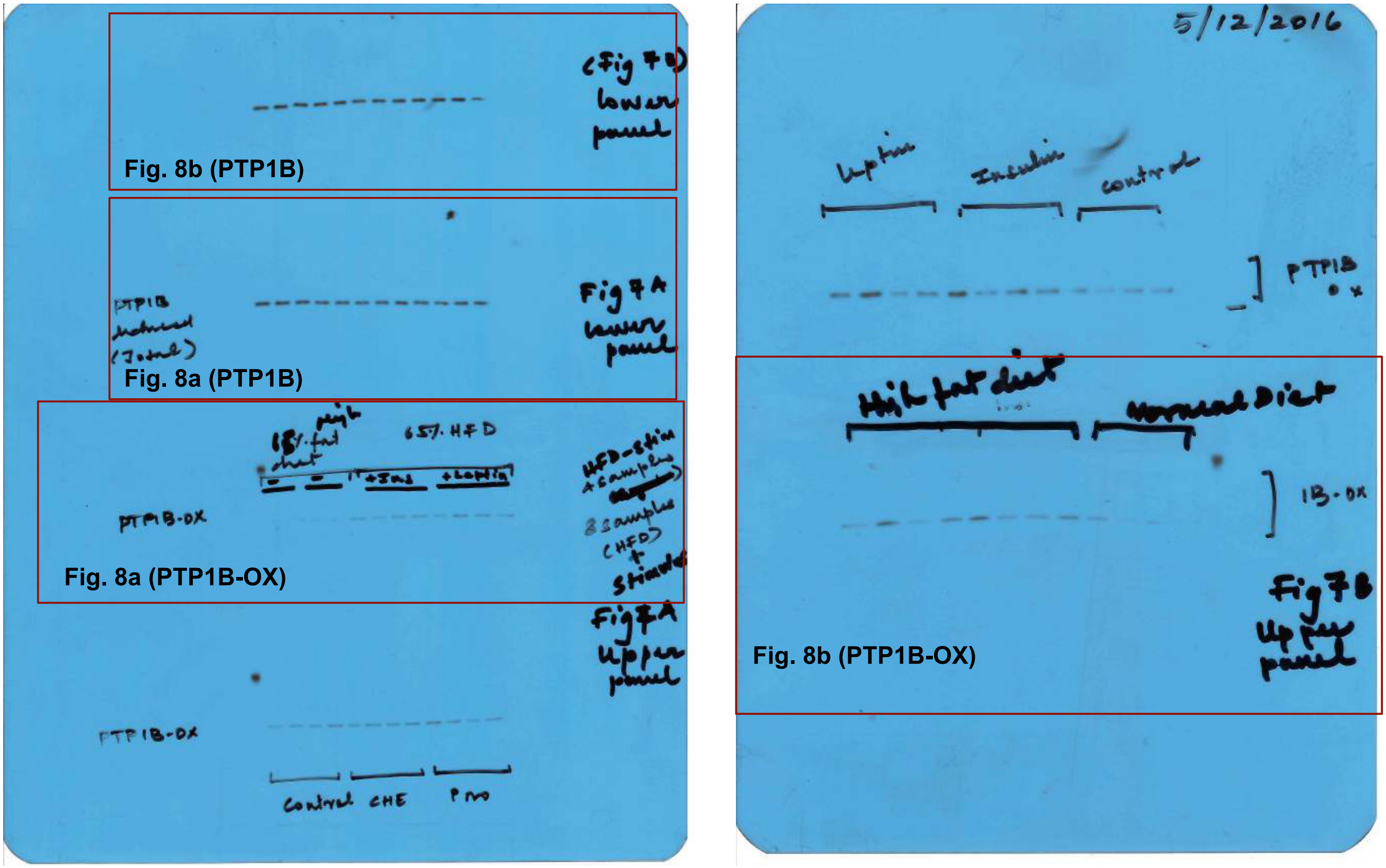

Uncropped immunoblot images from Figure 8. Shown are uncropped immunoblots presented in Fig. 8a & 8b. Images are labeled corresponding to their respective data from the manuscript, each individually denoted by a red box. Data are representative of three independent experiments.

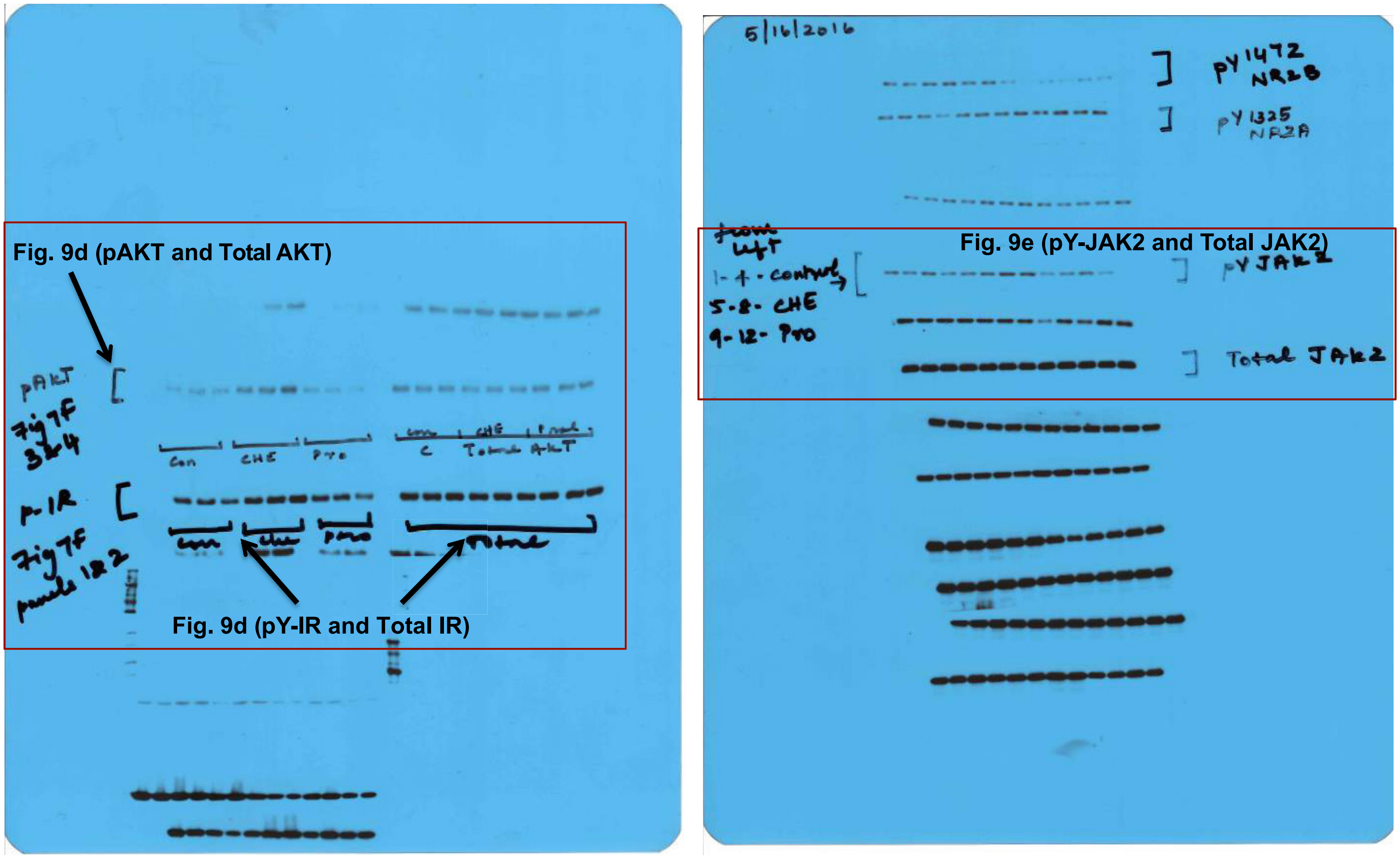

Uncropped immunoblot images from Figure 9. Shown are uncropped immunoblots presented in Fig. 9d & 9e. Images are labeled corresponding to their respective data from the manuscript, each individually denoted by a red box. Data are representative of three independent experiments.
